# Time-Course of Muscle Mass Loss, Damage, and Proteolysis in Gastrocnemius following Unloading and Reloading: Implications in Chronic Diseases

**DOI:** 10.1371/journal.pone.0164951

**Published:** 2016-10-28

**Authors:** Alba Chacon-Cabrera, Helena Lund-Palau, Joaquim Gea, Esther Barreiro

**Affiliations:** 1 Pulmonology Department-Muscle Wasting and Cachexia in Chronic Respiratory Diseases and Lung Cancer Research group, IMIM-Hospital del Mar, Parc de Salut Mar, Health and Experimental Sciences Department (CEXS), Universitat Pompeu Fabra (UPF), Barcelona Biomedical Research Park (PRBB), C/ Dr. Aiguader, 88, Barcelona, E-08003 Spain; 2 Centro de Investigación en Red de Enfermedades Respiratorias (CIBERES), Instituto de Salud Carlos III (ISCIII), Barcelona, Spain; University of Sydney, AUSTRALIA

## Abstract

**Background:**

Disuse muscle atrophy is a major comorbidity in patients with chronic diseases including cancer. We sought to explore the kinetics of molecular mechanisms shown to be involved in muscle mass loss throughout time in a mouse model of disuse muscle atrophy and recovery following immobilization.

**Methods:**

Body and muscle weights, grip strength, muscle phenotype (fiber type composition and morphometry and muscle structural alterations), proteolysis, contractile proteins, systemic troponin I, and mitochondrial content were assessed in gastrocnemius of mice exposed to periods (1, 2, 3, 7, 15 and 30 days) of non-invasive hindlimb immobilization (plastic splint, I cohorts) and in those exposed to reloading for different time-points (1, 3, 7, 15, and 30 days, R cohorts) following a seven-day period of immobilization. Groups of control animals were also used.

**Results:**

Compared to non-exposed controls, muscle weight, limb strength, slow- and fast-twitch cross-sectional areas, mtDNA/nDNA, and myosin content were decreased in mice of I cohorts, whereas tyrosine release, ubiquitin-proteasome activity, muscle injury and systemic troponin I levels were increased. Gastrocnemius reloading following splint removal improved muscle mass loss, strength, fiber atrophy, injury, myosin content, and mtDNA/nDNA, while reducing ubiquitin-proteasome activity and proteolysis.

**Conclusions:**

A consistent program of molecular and cellular events leading to reduced gastrocnemius muscle mass and mitochondrial content and reduced strength, enhanced proteolysis, and injury, was seen in this non-invasive mouse model of disuse muscle atrophy. Unloading of the muscle following removal of the splint significantly improved the alterations seen during unloading, characterized by a specific kinetic profile of molecular events involved in muscle regeneration. These findings have implications in patients with chronic diseases including cancer in whom physical activity may be severely compromised.

## Introduction

Muscle dysfunction and wasting are common comorbidities of chronic diseases such as cancer, critical illness, chronic heart failure and respiratory conditions, e.g. chronic obstructive pulmonary disease (COPD) [1–5]. Muscle mass loss and dysfunction negatively impact the patients’ exercise performance, thus severely limiting their quality of life and survival irrespective of the underlying condition [1–5]. Additionally, malnutrition and other metabolic disorders and aging may aggravate chronic disease-associated muscle wasting in patients. Therefore, these are serious comorbidities that need to be frequently overcome in actual clinical settings.

The most important players involved in the pathophysiology of muscle mass loss associated with respiratory and cardiac disorders and cancer are systemic inflammation, hypoxia, malnutrition, drugs, cigarette smoking, and deconditioning [3,4,6]. Prolonged bed rest and immobilization contribute to a great extent to muscle wasting in patients with chronic diseases. However, whether shorter periods of immobilization or reduced physical activity may equally lead to muscle mass loss and atrophy remains to be fully identified.

Several biological events and signaling pathways have been demonstrated to mediate muscle mass loss in patients and animal models of cachexia and muscle wasting [7–12]. For instance, oxidative stress, enhanced muscle proteolysis and ubiquitin-proteasome system, and epigenetic events were significantly upregulated in muscles of patients with COPD and lung cancer [11–15] and in respiratory and limb muscles of rodents with cancer-induced cachexia [8–10], chronic heart failure [16], and emphysema [17]. Furthermore, in models of disuse muscle atrophy following prolonged immobilization, oxidative stress [18,19], enhanced proteolysis [18,19], and reduced protein synthesis [20] were also shown to be involved in the process of muscle mass loss in several models.

Interestingly, short-term immobilization also reduced myofibrillar protein synthesis rates and anabolic resistance to protein ingestion in the quadriceps of healthy young humans [20]. Markers of ubiquitin-proteasome pathway and atrophy were also shown in the quadriceps of healthy elderly subjects following short-term immobilization [21]. Nonetheless, short periods of hindlimb unloading did not induce an accumulation of lipids or ceramide production in muscles of the animals [20,22]. Importantly, recovery of muscle mass loss may also be hampered by other factors, e.g. aging as recently shown in elderly subjects [23]. However, the precise time-course of recovery of muscle mass loss and function and underlying biological events following mechanical reloading remains to be thoroughly characterized.

On the basis of this, we sought to explore the kinetics of the molecular mechanisms shown to be involved in muscle mass loss throughout time in a mouse model of disuse muscle atrophy. Additionally, we also hypothesized that the kinetics of those molecular events may also vary over time during muscle recovery following a standardized immobilization period. Accordingly, our objectives were that in gastrocnemius of mice exposed to different periods (1, 2, 3, 7, 15 and 30 days) of non-invasive hindlimb immobilization and in those exposed to recovery (reloading) for different time-points (1, 3, 7, 15, and 30 days) after a seven-day period of immobilization, the following molecular events were explored: 1) muscle strength and structure and contractile proteins, 2) proteolysis and markers of ubiquitin-proteasome system, 3) markers of muscle anabolism, 4) mitochondrial biogenesis, and 5) systemic damage (blood troponin). The model of unilateral hindlimb immobilization has been previously validated in other investigations [24–26].

## Materials and Methods

See S1 File for detailed descriptions of all the study methodologies.

### Animal experiments

Female C57BL/6J mice (10 weeks old, weight ~20 g) were obtained from Harlan *Interfauna Ibérica SL* (Barcelona, Spain). Mice were kept under pathogen-free conditions in the animal house facility at Barcelona Biomedical Research Park (PRBB), with a 12:12 h light: dark cycle.

Mice were exposed to unilateral hindlimb immobilization as previously described to reproduce a non-invasive model of disuse muscle atrophy [24]. Briefly, the left hindlimb was shaved with clippers and was enveloped using surgical tape. The hindlimb was introduced in a 1.5 mL microcentrifuge tube with cover and bottom lids removed, while maintaining the foot in a plantar-flexed position to induce the maximal atrophy of the target limb muscle. Specific mouse restrainers were used in order to avoid anesthesia or sedation of the animals during application and removal of the splints. As the weight of the tube was approximately 0.6 g, it did not interfere with the usual mobility of the mice. In the study, the following control groups of mice were used for different purposes. Firstly, in order to assess potential differences in body weight and food intake, age-matched non-immobilized control mice were used for all the study groups (see below). Secondly, in order to explore potential differences in the variables muscle weight, tyrosine release, proteasome activities, mitochondrial content, muscle phenotype and morphometry, and structural abnormalities, the contralateral non-immobilized limb was used for these experiments. Thirdly, with the aim to evaluate potential differences in several markers of proteolysis, signaling pathways, and structural and functional proteins using immunoblotting, and blood troponin I levels using enzyme-linked immunosorbent assay (ELISA) a group of 30-day non-immobilized mice (period long enough to ensure potential differences if any) and a group of 7-day immobilized rodents, were used as the control group of the immobilization and recovery time-cohorts, respectively. Mice maintained normally their physical activity throughout the study protocol except for the immobilized hindlimb (S1 Video).

As shown in Figure A in S1 File, three different approaches were taken in the investigation: 1) non-immobilization group, 2) immobilization time-cohorts (I groups), and 3) recovery time-cohorts (R groups), in which the left hindlimb of the mice was immobilized for seven consecutive days, time at which the splint was removed to let the animals move freely in their cages to evaluate muscle recovery at different time-points. Afterwards, animals were randomly assigned to the following groups (N = 10/group): 1) 30-day non-immobilized control group; 2) mice immobilized for one day (1-day I); 3) mice immobilized for two days (2-day I); 4) mice immobilized for three days (3-day I); 5) mice immobilized for seven days (7-day I); 6) mice immobilized for fifteen days (15-day I); 7) mice immobilized for thirty days (30-day I); 8) mice exposed to seven days of unilateral hindlimb immobilization followed by one day recovery (1-day R); 9) mice exposed to seven days of unilateral hindlimb immobilization followed by three days recovery (3-day R); 10) mice exposed to seven days of unilateral hindlimb immobilization followed by seven days recovery (7-day R); 11) mice exposed to seven days of unilateral hindlimb immobilization followed by fifteen days recovery (15-day R); 12) mice exposed to seven days of unilateral hindlimb immobilization followed by thirty days recovery (30-day R).

All animal experiments were conducted in the animal facilities at *Parc de Recerca Biomèdica de Barcelona* (PRBB). This controlled study was designed in accordance with the ethical standards on animal experimentation (EU 2010/63 CEE, *Real Decreto* 53/2013 BOE 34, Spain) at PRBB and the Helsinki convention for the use and care of animals. Ethical approval was obtained by the Animal Research Committee (Animal welfare department in Catalonia, EBP-13-1485).

#### *In vivo* measurements in the mice

In all the study animals, body weight and food intake were measured at every time-point, and food and water were supplied ad libitum for the entire duration of the immobilization or recovery periods. In all mice, limb strength was determined on day 0, day 30 (non-immobilized controls), and right at the end of each immobilization or recovery time-points (as described above) using a grip strength meter (Bioseb, Vitrolles Cedex, France) following previously published methodologies, in which grip strength was also the end-point parameter in the different experimental models [8,9,16,27]. In all mice, the four limbs equally contributed to the maneuver of grip strength (Figure B in S1 File, and S2 Video). In all the animals, limb strength gain was calculated as the percentage of the measurements performed at the end of the study period with respect to the same measurements obtained at baseline (grip strength at the end of the study period–grip strength on day 0)/ grip strength on day 0 x 100).

#### Sacrifice and sample collection

Mice from all the experimental groups were sacrificed after the corresponding immobilization or recovery time-cohorts, or after 30 days (non-immobilized control group). Each mouse was previously inoculated intraperitoneally with 0.1 mL sodium pentobarbital (60 mg/Kg). In all cases, the pedal and blink reflexes were evaluated in order to verify total anesthetic depth. The following samples were obtained from all the animals at the time of sacrifice: blood and gastrocnemius muscle. Animals were sacrificed after collecting blood and gastrocnemius samples including the diaphragm, which induced the immediate death of the mice in all study groups. Blood samples, which were obtained through puncture of the saphenous vein, were centrifuged at 1,200 rpm for 15 minutes to yield plasma. Muscle samples were snap-frozen in liquid nitrogen to be thereafter stored frozen at -80°C to be further used for the molecular analyses. Moreover, another fragment of the muscle specimens was paraffin-embedded to be used for the assessment of muscle structure abnormalities and fiber type composition and morphometry.

### Biological analyses

#### ELISA plasma skeletal muscle troponin-I levels

Skeletal muscle troponin-I levels were quantified in plasma of the following groups of animals: 1-day I, 3-day I, 7-day I, 15-day I, 30-day I, 1-day R, 3-day R, 15-day R, 30-day R, and the 30-day non-immobilized controls using a specific sandwich ELISA kit (Life Diagnostics Inc., West Chester, PA, USA) as previously shown [28–31].

#### Muscle DNA isolation

Total DNA, including mitochondrial and nuclear DNA was isolated from gastrocnemius muscle of all mouse experimental groups using QIAmp DNA Mini Kit (QiAgen, GmbH, Germany), following the manufacturer’s protocol of DNA purification from tissues, and without the use of RNase A, as previously shown [32]. Total DNA obtained from muscles was quantified using a spectrophotometer (Thermo Scientific, Waltham, MA, USA).

#### Absolute quantification by real-time PCR of DNAs

Mitochondrial DNA (mtDNA) copy numbers were estimated through the quantification of the mtDNA to nuclear DNA (nDNA) ratio (mtDNA/nDNA). The content of mtDNA was determined using singleplex (amplifying one target sequence per well) qRT-PCR analyses, and corrected by the simultaneous measurements of a single copy of nuclear angiogenin-1 (ANG1) gene. The mtDNA 16S rRNA primers and TaqMan probe were used as also previously reported [33].

#### Immunoblotting of 1D electrophoresis

Protein levels of the different molecular markers analyzed in the study were explored by means of immunoblotting procedures as previously described [8,9,16]. Briefly, frozen muscle samples from the gastrocnemius muscle of all mouse experimental groups were homogenized in a buffer containing 50 mM 4-(2-hydroxyethyl)-1-piperazineethanesulfonic acid (HEPES), 150 mM NaCl, 100 mM NaF, 10 mM Na pyrophosphate, 5 mM ethylenediaminetetraacetic acid (EDTA), 0.5% Triton-X, 2 micrograms/mL leupeptin, 100 micrograms/mL phenylmethanesulfonyl fluoride (PMSF), 2 micrograms/mL aprotinin and 10 micrograms/mL pepstatin A. Moreover, myofibrillar proteins were also isolated in order to identify levels of actin and myosin heavy chain (MyHC) as previously reported [8,16,34,35]. Two independent sets of immunoblots were conducted, in which muscle homogenates from immobilized and recovery groups of mice were run separately. Four fresh 10-well mini-gels were always simultaneously loaded for each of the study antigens analyzed using immunoblotting.

Proteins were then separated by electrophoresis, transferred to polyvinylidene difluoride (PVDF) membranes, blocked with bovine serum albumin and incubated overnight with selective primary antibodies. Protein levels of signaling pathways, proteolysis, anabolism and muscle contractile proteins were identified in the gastrocnemius using specific primary antibodies: α-actin (anti-α-sarcomeric actin antibody, clone 5C5, Sigma-Aldrich), myosin heavy chain (anti-MyHC antibody, clone A4.1025, Upstate-Millipore), RAC-alpha serine/threonine-protein kinase (Akt) and p-Akt (anti-Akt and anti-p-Akt antibodies, Cell Signaling Technology), p70 S6 kinase (p70S6K) and p-p70S6K (anti- p70S6K and anti-p-p70S6K antibodies, Cell Signaling Technology), total ubiquitinated proteins (anti-ubiquitinated proteins antibody, Boston Biochem), 20S proteasome subunit C8 (anti-C8 antibody, Biomol), tripartite motif containing 32 (TRIM32) (anti-TRIM32 antibody, Santa Cruz Biotechnology), ubiquitin-ligase atrogin-1 (anti-atrogin-1 antibody, Santa Cruz Biotechnology), ubiquitin-ligase muscle ring finger (MURF)-1 (anti-MURF-1 antibody, Everest Biotech), growth differentiation factor 15 (GDF-15) (anti-GDF15 antibody, Santa Cruz Biotechnology) and glyceraldehyde-3-phosphate dehydrogenase (GAPDH) (anti-GAPDH antibody, Santa Cruz Biotechnology). Antigens from all samples were detected with horseradish peroxidase (HRP)-conjugated secondary antibodies and a chemiluminescence kit. For each of the antigens, samples from the different groups were always detected in the same picture under identical exposure times. The specificity of the different antibodies was confirmed by omission of the primary antibody, and incubation of the membranes only with secondary antibodies.

PVDF membranes were scanned with the Molecular Imager Chemidoc XRS System (Bio–Rad Laboratories, Hercules, CA, USA) using the software Quantity One version 4.6.5 (Bio–Rad Laboratories). Optical densities of specific proteins were quantified using the software Image Lab version 2.0.1 (Bio-Rad Laboratories). Final optical densities obtained in each specific group of subjects and muscle corresponded to the mean values of the different samples (lanes) of each of the antigens studied. In order to validate equal protein loading among various lanes, the glycolytic enzyme GAPDH was used as the protein loading controls in all the immunoblots.

Standard stripping methodologies were employed when detection of the antigens required the loading of a relatively greater amount of total muscle protein. Membranes were stripped of primary and secondary antibodies after a 30-minute wash with a specific stripping solution [25 mM glycine, pH 2.0 and 1% sodium dodecyl sulfate (SDS)] followed by two consecutive 10-minute washes containing phosphate buffered saline with tween (PBST) at room temperature. Membranes were blocked with bovine serum albumin (BSA) and reincubated with primary and secondary antibodies following the procedures described above.

#### Protein catabolism

Protein degradation in muscles was explored on the basis of the rate of production of free tyrosine from tissue proteins as previously described [8,16,36,37].

#### Proteasome activities

The first step included the isolation of the proteasome, in which previously published procedures were followed [16,38].

Chymotrypsin-like activity. In the gastrocnemius muscle of all study animals, chymotrypsin-like activity was evaluated according to the cleavage of the fluorescent substrate Suc-LLVY-AMC (Biomol International, Plymouth Meeting, PA, EEUU) and the generation of a fluorogenic product, the methyl coumarilamide (AMC) following previously published methodologies [16,38].

#### Immunohistochemistry

On 3-micrometer muscle paraffin-embedded sections from gastrocnemius muscle of all study groups, MyHC-I and–II isoforms were identified using anti-MyHC-I (clone MHC, Biogenesis Inc.) and anti-MyHC-II antibodies (clone MY-32, Sigma-Aldrich), respectively, as published elsewhere [8,9,16].

#### Muscle structure abnormalities

The area fraction of normal and abnormal muscle was evaluated on 3-micrometer paraffin-embedded sections of the gastrocnemius of all study groups muscles following previously published methodologies [8,9,16]. The identified items in the muscle cross-sections are shown in Figure C in S1 File.

### Statistical analysis

Normality of the study variables were checked using the Shapiro-Wilk test. Physiological, structural, and molecular results were expressed as mean (standard deviation). The following statistical approaches were used in the study for different purposes. Firstly, total body weight and food intake of mice from each experimental group (I and R cohorts) were compared with their corresponding age-matched non-immobilized controls using the unpaired Student’s T-test. For each pair, a level of significance of *P*≤ 0.05 was established.

Secondly, deltas of the difference of mean values of the results obtained in the gastrocnemius of the immobilized hindlimb with respect to those of the contralateral non-immobilized hindlimb, were also calculated in each animal from both cohorts (I and R), and from 30-day non-immobilized animals for another set of comparisons for the following variables: muscle weight, tyrosine release, proteasome activities, mitochondrial content, muscle phenotype and morphometry, and structural abnormalities. In this case, results are expressed as mean delta (standard deviation). Deltas obtained from each group were compared as follows: 1) deltas from mice of the immobilized cohorts (I groups) versus deltas of 30-day non-immobilized controls and 2) deltas from mice of the recovery cohorts (R groups) versus deltas of mice that were exposed to seven days immobilization (7-day I, control group). Potential significant differences were assessed using one-way analysis of variance (ANOVA) with *Dunnett’s post hoc* analysis to adjust for multiple comparisons among the study groups. A level of significance of P≤ 0.05 was established.

Thirdly, for the following variables: 20S proteasome C8, ubiquitin, E3-ligases, GDF15, MyHC, actin, Akt, P70S6K, and troponin I, comparisons were made between each group of mice from the different time-points of the I cohorts and the 30-day non-immobilized animals, and from the different time-points of the R cohorts compared with 7-day I. The following comparisons were performed to explore potential differences among the groups: 1) mice from the immobilized cohorts (I groups) versus the 30-day non-immobilized controls and 2) animals from the recovery cohorts (R groups) versus animals immobilized for 7 days (7-day I, control group). In these comparisons, potential significant differences were assessed using one-way analysis of variance (ANOVA) with *Dunnett’s post hoc* analysis to adjust for multiple comparisons among the study groups. A level of significance of *P*≤ 0.05 was established.

The sample size chosen was based on previous studies [8,9,16,17], where very similar approaches were employed. In addition, statistical power was calculated using specific software (StudySize 2.0, CreoStat HB, Frolunda, Sweden). Limb strength gain and changes in myofiber cross sectional area were selected as the target variables to estimate the statistical power in the study. On the basis of a standard power statistics established at a minimum of 80% and assuming an alpha error of 0.05, the statistical power was sufficiently high to detect a minimum difference of 25 points of delta in limb strength gain and 300 points of delta in myofiber cross sectional area respectively, among the different study groups for the given sample size and standard deviations.

## Results

### Physiological characteristics of the study animals

#### Immobilization

**Fig 1:**
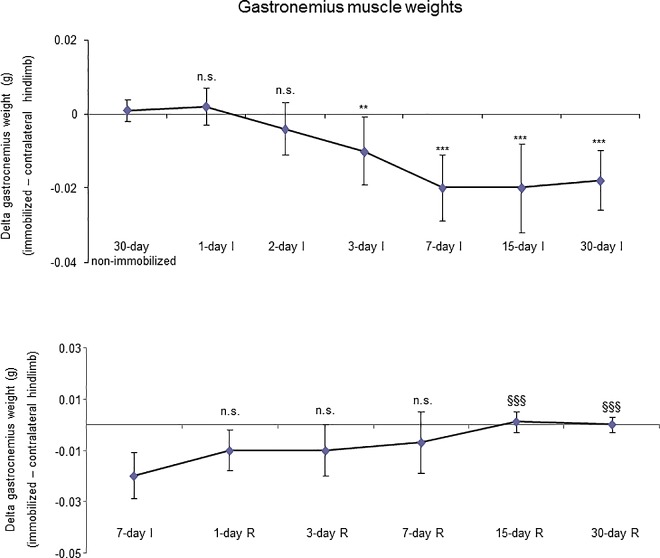
Delta gastrocnemius muscle weights of all the groups in both study cohorts. Mean values and standard deviation of delta of the variable gastrocnemius weight in the immobilization (top panel) and recovery (bottom panel) cohorts of mice. Definition of abbreviations: g, grams; I, immobilization; R, recovery. Statistical significance is represented as follows: **, p≤0.01, ***, p≤0.001, and n.s., non-significant differences between any of the immobilized animals and the non-immobilized controls; §§§, p≤0.001, and n.s., non-significant differences between any of the R groups of animals and the 7-day I mice.

**Fig 2:**
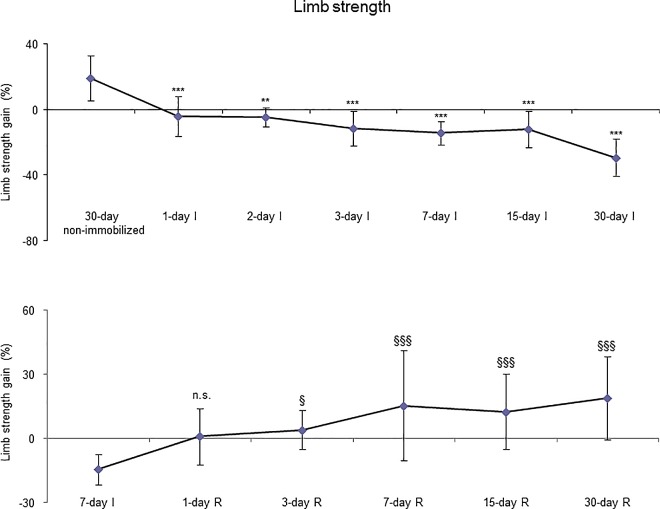
Limb strength gain of all the groups in both study cohorts. Mean values and standard deviation of limb strength gain of the immobilization (top panel) and recovery (bottom panel) cohorts of mice. Definition of abbreviations: I, immobilization; R, recovery. Statistical significance is represented as follows: **, p≤0.01, and ***, p≤0.001 between any of the immobilized animals and the non-immobilized controls; §, p≤0.05, §§§, p≤0.001, and n.s., non-significant differences between any of the R groups of animals and the 7-day I mice.

As shown in Figure D (top panel) in S1 File, and in Table A in S1 File, total body weight and food intake did not differ between animals in the I cohorts and their age-matched non-immobilized controls. The delta change of gastrocnemius muscle weight (immobilized versus contralateral non-immobilized hindlimb) was significantly reduced in 3-day I, 7-day I, 15-day I and 30-day I animals compared to non-immobilized controls (Fig 1, top panel). A statistically significant decrease in limb strength gain was observed in all groups of the I cohorts compared to non-immobilized mice (Fig 2, top panel).

#### Recovery

Total body weight (Figure D, bottom panel, in S1 File) and food intake (Table A in S1 File) did not differ among the study groups. Delta changes of gastrocnemius muscle weights were significantly increased in 15- and 30-day R groups compared to the control mice (7-day I animals, Fig 1, bottom panel). Limb strength gain significantly improved in 3-, 7-, 15- and 30-day R cohorts of mice compared to the controls (7-day I mice, Fig 2, bottom panel).

### Muscle structure characteristics

#### Immobilization

**Fig 3:**
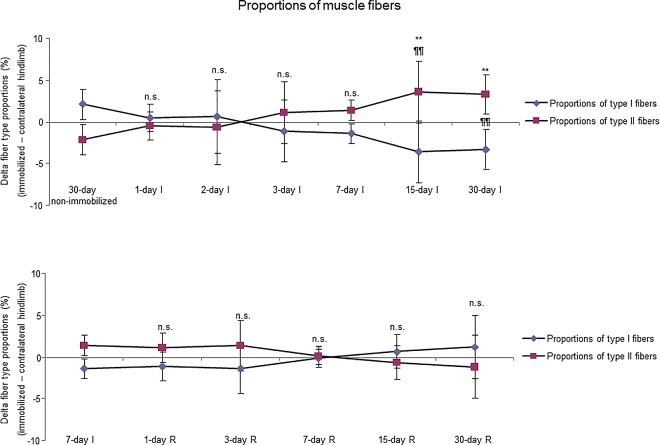
Delta of muscle fibers proportions of all the groups in both study cohorts. Mean values and standard deviation of delta of the variables type I and type II fibers proportions of the immobilization (top panel) and recovery (bottom panel) cohorts of mice. Definition of abbreviations: I, immobilization; R, recovery. Statistical significance is represented as follows: **, p≤0.01, and n.s., non-significant differences in type I fibers proportions between any of the immobilized animals and the non-immobilized controls; ¶¶, p≤0.01, and n.s., non-significant differences in type II fibers proportions between any of the immobilized animals and the non-immobilized controls; n.s., non-significant differences in either type I or type II fibers proportions between any of the R groups of animals and the 7-day I mice.

**Fig 4:**
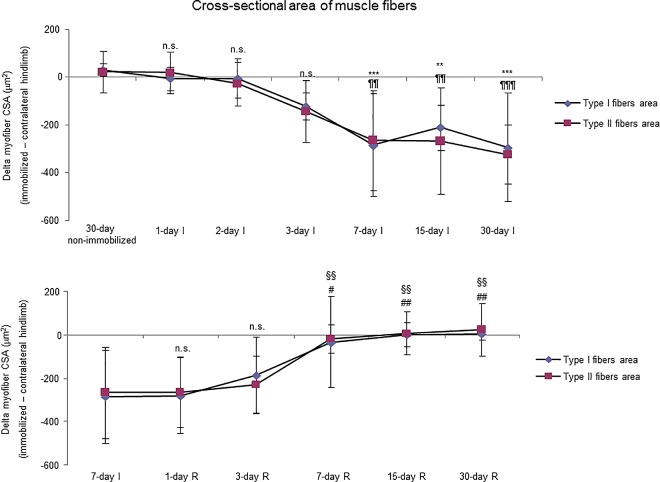
Delta of the slow- and fast-twitch fibers cross-sectional area of all the groups in both study cohorts. Mean values and standard deviation of delta of the variables fiber type I and type II cross sectional areas of the immobilization (top panel) and recovery (bottom panel) cohorts of mice. Definition of abbreviations: μm^2^, square micrometers; I, immobilization; R, recovery. Statistical significance is represented as follows: **, p≤0.01, ***, p≤0.001, and n.s., non-significant differences in type I cross-sectional area between any of the immobilized animals and the non-immobilized controls; ¶¶, p≤0.01, ¶¶¶, p≤0.001, and n.s., non-significant differences in type II cross-sectional area between any of the immobilized animals and the non-immobilized controls; §§, p≤0.01, and n.s., non-significant differences in type I cross-sectional area between any of the R groups of animals and the 7-day I mice; #, p≤0.05, ##, p≤0.01, and n.s., non-significant differences in type II cross-sectional area between any of the R groups of animals and the 7-day I mice.

**Fig 5:**
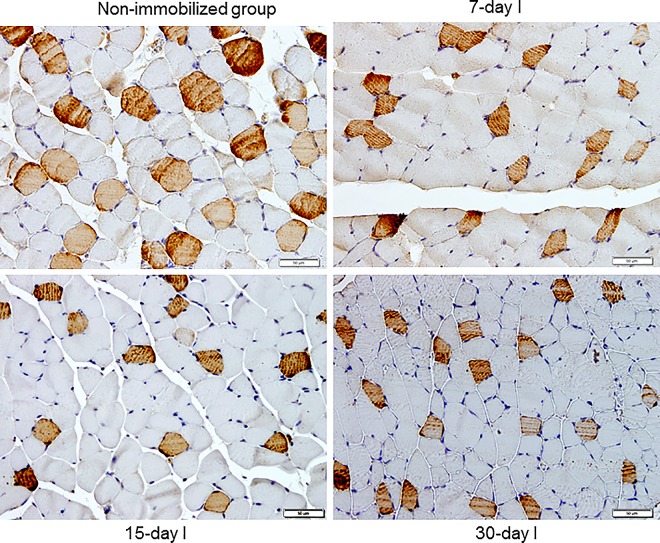
Gastrocnemius slow- and fast-twitch fibers cross-sectional area was reduced by immobilization. Representative examples of the gastrocnemius muscle in animals of the 7-, 15-, and 30-day immobilized cohorts, and non-immobilized control group. Myofibers positively stained with the anti-MyHC type I antibody are stained in brown color (x 400). Type II fibers were not stained (white color).

**Fig 6:**
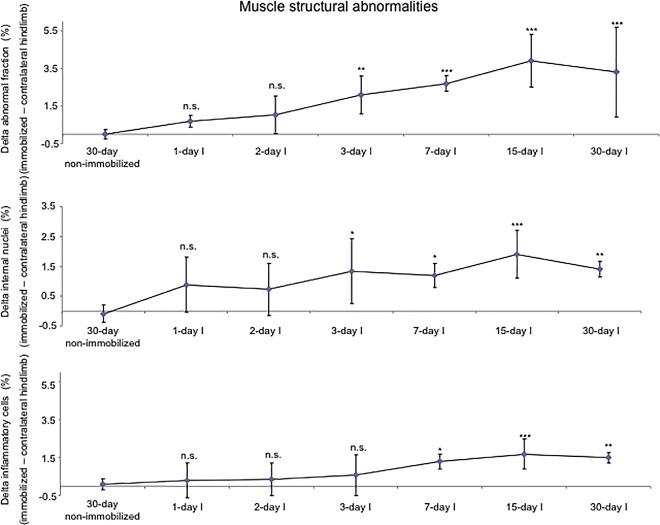
Delta of muscle structural abnormalities of all the groups of the immobilization cohorts. Mean values and standard deviation of deltas of the variables abnormal fraction (top panel), internal nuclei (middle panel), and inflammatory cells (bottom panel) in the gastrocnemius of the immobilization cohorts of animals. Definition of abbreviations: I, immobilization; R, recovery. Statistical significance is represented as follows: *, p≤0.05, **, p≤0.01, ***, p≤0.001, and n.s., non-significant differences between any of the immobilized animals and the non-immobilized controls.

Proportions of slow-twitch muscle fibers were significantly reduced, while those of fast-twitch were increased in 15- and 30-day I study cohorts compared to the controls (non-immobilized mice, Fig 3, top panel, and Figure E in S1 File). The sizes of both slow- and fast-twitch fibers were significantly reduced in the gastrocnemius of 7-, 15-, and 30-day I mice compared to the controls (non-immobilized animals, Fig 4, top panel, Fig 5, and Figure E in S1 File). Compared to non-immobilized animals, proportions of total abnormal muscle fraction and internal nuclei counts were increased in the gastrocnemius of 3-, 7-, 15-, and 30-day I study cohorts (Fig 6, top and middle panels). Inflammatory cell counts were greater in the gastrocnemius of 7-, 15-, and 30-day I animals than in the controls (non-immobilized mice, Fig 6, bottom panel).

#### Recovery

**Fig 7:**
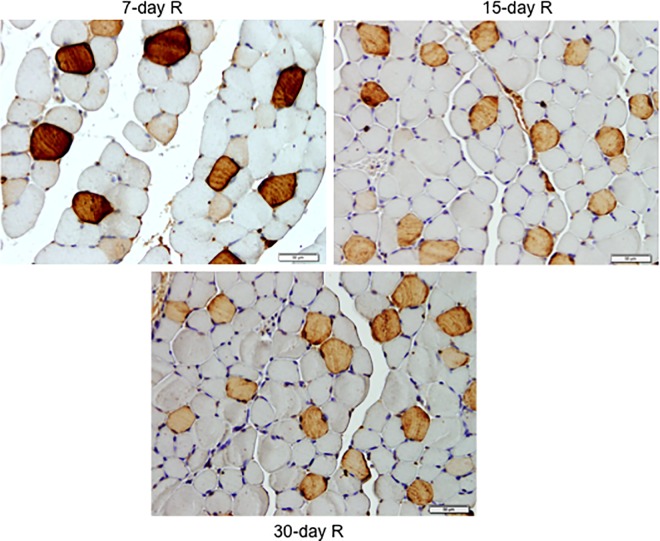
Gastrocnemius slow- and fast-twitch fibers cross-sectional area improved after the recovery period. Representative examples of the gastrocnemius muscle in animals of the 7-, 15-, and 30-day recovery cohorts. Myofibers positively stained with the anti-MyHC type I antibody are stained in brown color (x 400). Type II fibers were not stained (white color).

**Fig 8:**
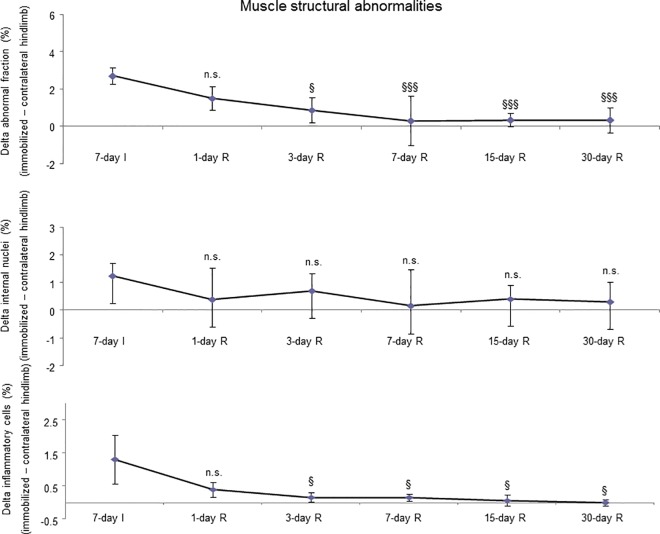
Delta of muscle structural abnormalities of all the groups of the recovery cohorts. Mean values and standard deviation of deltas of the variables abnormal fraction (top panel), internal nuclei (middle panel), and inflammatory cells (bottom panel) in the gastrocnemius of the recovery cohorts of rodents. Definition of abbreviations: I, immobilization; R, recovery. Statistical significance is represented as follows: §, p≤0.05, §§§, p≤0.001, and n.s., non-significant differences between any of the R groups of animals and the 7-day I mice.

No significant differences were seen in any of the R study cohorts regarding the proportions of type I or type II fibers (Fig 3, bottom panel, and Figure F in S1 File). The size of both fast- and slow-twitch fibers significantly increased in the gastrocnemius of 7-, 15-, and 30-day R study cohorts compared to the controls (7-day immobilized animals, Fig 4, bottom panel, Fig 7, and Figure F in S1 File). Proportions of total abnormal muscle fraction and inflammatory cell counts significantly reduced in the gastrocnemius of 3-, 7-, 15-, and 30-day R study cohorts compared to the controls (7-day I mice, Fig 8, top and bottom panels). Internal nuclei counts did not significantly differ among the study groups (Fig 8, middle panel).

### Tyrosine release

#### Immobilization

**Fig 9:**
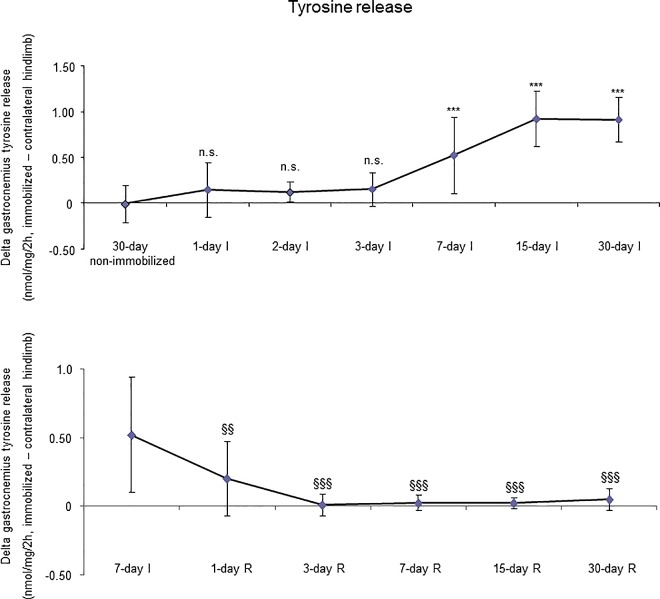
Delta of gastrocnemius tyrosine release of all the groups in both study cohorts. Mean values and standard deviation of delta of the variable tyrosine release (nmol/mg/2h) in the gastrocnemius of the immobilization (top panel) and recovery (bottom panel) cohorts of mice. Definition of abbreviations: nmol, nanomol; mg, milligram; h, hour; I, immobilization; R, recovery. Statistical significance is represented as follows: ***, p≤0.001, and n.s., non-significant differences between any of the immobilized animals and the non-immobilized controls; §§, p≤0.01, and §§§, p≤0.001 between any of the R groups of animals and the 7-day I mice.

Tyrosine release significantly increased in the gastrocnemius of 7-, 15-, and 30-day groups compared to the controls (non-immobilized animals, Fig 9, top panel).

#### Recovery

Tyrosine release levels significantly decreased in the gastrocnemius of all R study cohorts of mice compared to the controls (7-day I mice, Fig 9, bottom panel).

### Proteolytic markers

#### Immobilization

**Fig 10:**
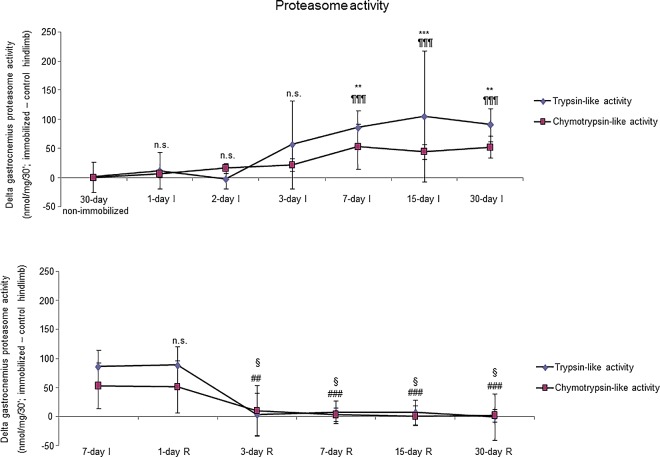
Delta of gastrocnemius proteasome trypsin-like and chymotrypsin-like activities of all the groups in both study cohorts. Mean values and standard deviation of delta of the variables trypsin-like and chymotrypsin-like activities (nmol/mg/30’) in the gastrocnemius of the immobilization (top panel) and recovery (bottom panel) cohorts of mice. Definition of abbreviations: nmol, nanomol; mg, milligram; I, immobilization; R, recovery. Statistical significance is represented as follows: **, p≤0.01, ***, p≤0.001 and n.s., non-significant differences in trypsin-like activity between any of the immobilized animals and the non-immobilized controls; ¶¶¶, p≤0.001, and n.s., non-significant differences in chymotrypsin-like activity between any of the immobilized animals and the non-immobilized controls; §, p≤0.05, and n.s., non-significant differences in trypsin-like activity between any of the R groups of animals and the 7-day I mice; ##, p≤0.01, ###, p≤0.001, and n.s., non-significant differences in chymotrypsin-like activity between any of the R groups of animals and the 7-day I mice.

**Fig 11:**
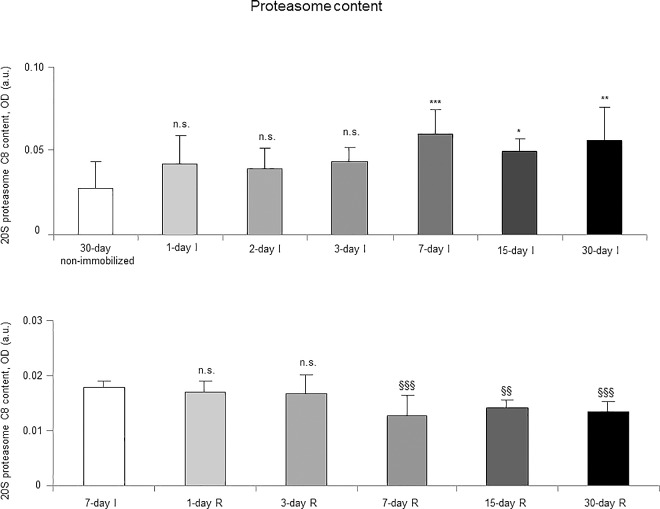
Gastrocnemius 20S proteasome C8 content of all the groups in both study cohorts. Mean values and standard deviation of 20S proteasome C8 subunit content in the gastrocnemius of the immobilization (top panel) and recovery (bottom panel) cohorts of mice, as measured by optical densities in arbitrary units (OD, a.u.). Definition of abbreviations: OD, optical densities; a.u., arbitrary units; I, immobilization; R, recovery. Statistical significance is represented as follows: *, p≤0.05, **, p≤0.01, ***, p≤0.001, and n.s., non-significant differences between any of the immobilized animals and the non-immobilized controls; §§, p≤0.01, §§§, p≤0.001, and n.s., non-significant differences between any of the R groups of animals and the 7-day I mice. Note that as arbitrary units were used for the measurement of the optical densities in each set of immunoblots (see methods), scale bars in the ordinate axes differ in the graphs of the immobilization groups from those of the recovery cohorts.

**Fig 12:**
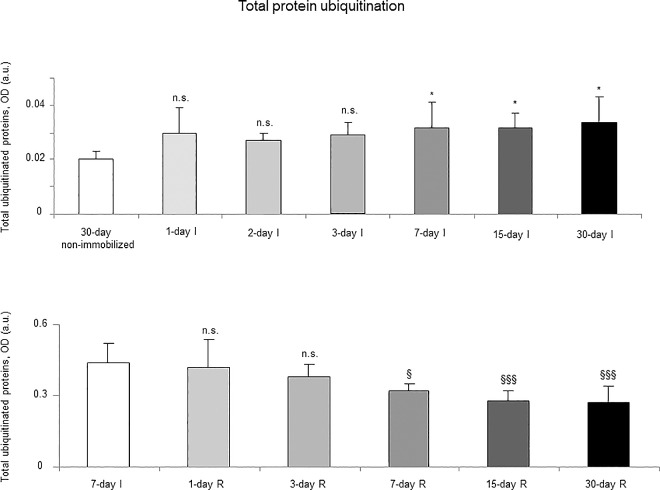
Total protein ubiquitination content of all the groups in both study cohorts. Mean values and standard deviation of total ubiquitinated proteins in the gastrocnemius of the immobilization (top panel) and recovery (bottom panel) cohorts of mice, as measured by optical densities in arbitrary units (OD, a.u.). Definition of abbreviations: OD, optical densities; a.u., arbitrary units; I, immobilization; R, recovery. Statistical significance is represented as follows: *, p≤0.05, and n.s., non-significant differences between any of the immobilized animals and the non-immobilized controls; §, p≤0.05, §§§, p≤0.001, and n.s., non-significant differences between any of the R groups of animals and the 7-day I mice. Note that as arbitrary units were used for the measurement of the optical densities in each set of immunoblots (see methods), scale bars in the ordinate axes differ in the graphs of the immobilization groups from those of the recovery cohorts.

**Fig 13:**
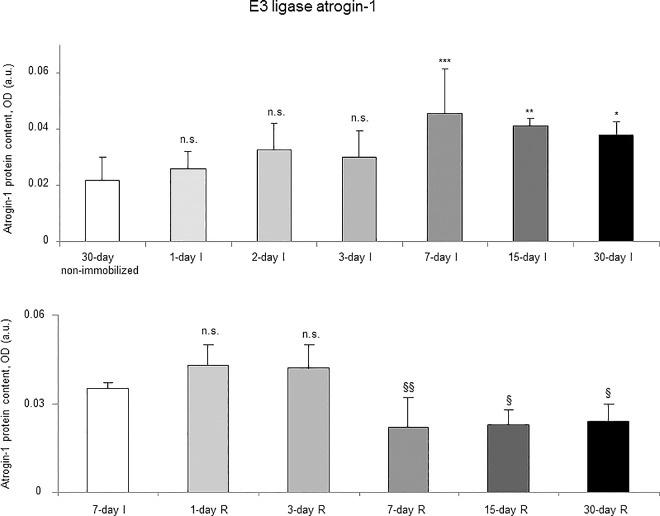
E3 ligase atrogin-1 content of all the groups in both study cohorts. Mean values and standard deviation of atrogin-1 protein content in the gastrocnemius of the immobilization (top panel) and recovery (bottom panel) cohorts of mice, as measured by optical densities in arbitrary units (OD, a.u.). Definition of abbreviations: OD, optical densities; a.u., arbitrary units; I, immobilization; R, recovery. Statistical significance is represented as follows: *, p≤0.05, **, p≤0.01, ***, p≤0.001, and n.s., non-significant differences between any of the immobilized animals and the non-immobilized controls; §, p≤0.05, §§, p≤0.01, and n.s., non-significant differences between any of the R groups of animals and the 7-day I mice. Note that as arbitrary units were used for the measurement of the optical densities in each set of immunoblots (see methods), scale bars in the ordinate axes differ in the graphs of the immobilization groups from those of the recovery cohorts.

**Fig 14:**
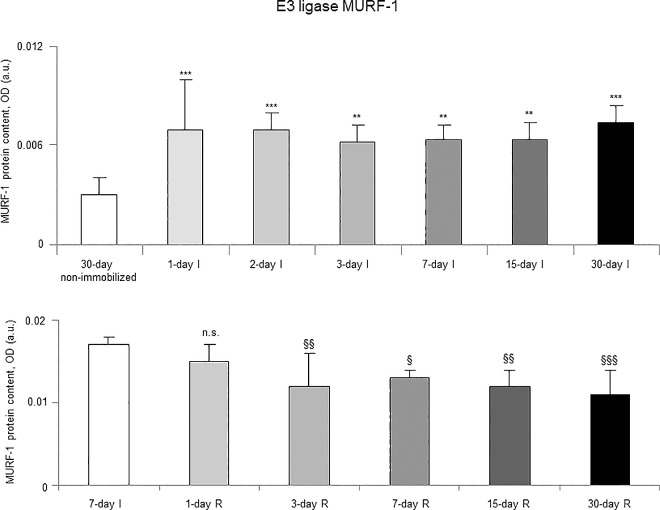
E3 ligase MURF-1 content of all the groups in both study cohorts. Mean values and standard deviation of MURF-1 protein content in the gastrocnemius of the immobilization (top panel) and recovery (bottom panel) cohorts of mice, as measured by optical densities in arbitrary units (OD, a.u.). Definition of abbreviations: MURF-1, muscle ring finger protein 1; OD, optical densities; a.u., arbitrary units; I, immobilization; R, recovery. Statistical significance is represented as follows: **, p≤0.01, ***, p≤0.001, and n.s., non-significant differences between any of the immobilized animals and the non-immobilized controls; §, p≤0.05, §§, p≤0.01, §§§, p≤0.001, and n.s., non-significant differences between any of the R groups of animals and the 7-day I mice. Note that as arbitrary units were used for the measurement of the optical densities in each set of immunoblots (see methods), scale bars in the ordinate axes differ in the graphs of the immobilization groups from those of the recovery cohorts.

**Fig 15:**
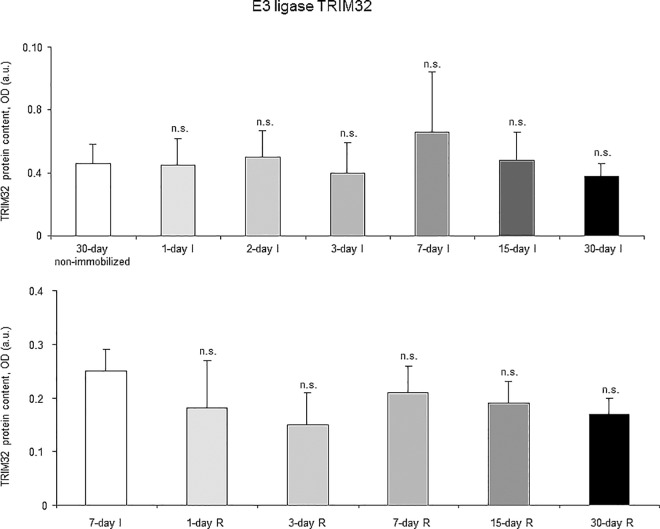
E3 ligase TRIM32 content of all the groups in both study cohorts. Mean values and standard deviation of TRIM32 protein content in the gastrocnemius of the immobilization (top panel) and recovery (bottom panel) cohorts of mice, as measured by optical densities in arbitrary units (OD, a.u.). Definition of abbreviations: TRIM32, tripartite motif containing 32; OD, optical densities; a.u., arbitrary units; I, immobilization; R, recovery. Statistical significance is represented as follows: n.s., non-significant differences between any of the immobilized animals and the non-immobilized controls; n.s., non-significant differences between any of the R groups of animals and the 7-day I mice. Note that as arbitrary units were used for the measurement of the optical densities in each set of immunoblots (see methods), scale bars in the ordinate axes differ in the graphs of the immobilization groups from those of the recovery cohorts.

**Fig 16:**
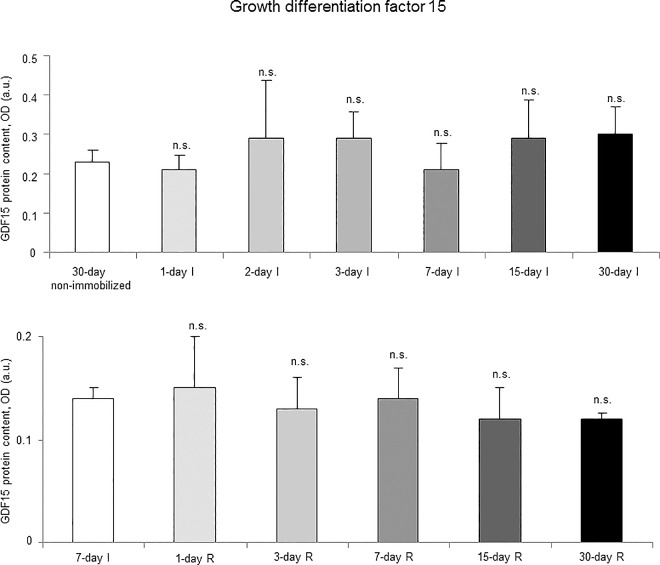
GDF15 content of all the groups in both study cohorts. Mean values and standard deviation of GDF15 protein content in the gastrocnemius of the immobilization (top panel) and recovery (bottom panel) cohorts of mice, as measured by optical densities in arbitrary units (OD, a.u.). Definition of abbreviations: GDF15, growth differentiation factor 15; OD, optical densities; a.u., arbitrary units; I, immobilization; R, recovery. Statistical significance is represented as follows: n.s., non-significant differences between any of the immobilized animals and the non-immobilized controls; n.s., non-significant differences between any of the R groups of animals and the 7-day I mice. Note that as arbitrary units were used for the measurement of the optical densities in each set of immunoblots (see methods), scale bars in the ordinate axes differ in the graphs of the immobilization groups from those of the recovery cohorts.

Proteasome trypsine-like and chymotrypsine-like activities significantly increased in 7-, 15- and 30-day I study cohorts compared to the controls (non-immobilized animals, Fig 10, top panels). Levels of 20S proteasome subunit C8, total protein ubiquitination, and atrogin-1 were significantly higher in the gastrocnemius of 7-, 15-, and 30-day I study cohorts of animals than in the controls (non-immobilized mice, Figs 11–13, top panels, and Figure G in S1 File). Protein content of E3 ligase MURF-1 increased in the gastrocnemius of all I study cohorts of mice compared to the controls (non-immobilized animals, Fig 14, top panel, and Figure G in S1 File). Muscle protein levels of TRIM32 and GDF15 did not significantly differ among study groups in the I cohorts (Figs 15 and 16, top panels, and Figure G in S1 File).

#### Recovery

Proteasome trypsine-like and chymotrypsine-like activities were significantly reduced in 3-, 7-, 15-, and 30-day R study cohorts of mice compared to controls (7-day I animals, Fig 10, bottom panels). Protein levels of 20S proteasome subunit C8, total protein ubiquitination, and atrogin-1 were significantly lower in the gastrocnemius of 7-, 15-, and 30-day groups of the R cohorts than in the controls (7-day I mice, Figs 11–13, bottom panels, and Figure H in S1 File). Levels of MURF-1 significantly decreased in the gastrocnemius of 3-, 7-, 15-, and 30-day study groups in the R cohorts compared to controls (7-day I mice, Fig 14, bottom panel, and Figure H in S1 File). Muscle protein levels of TRIM32 and GDF15 did not significantly differ among the study groups in the R cohorts (Figs 15 and 16, bottom panels, and Figure H in S1 File).

### Muscle structural proteins

#### Immobilization

**Fig 17:**
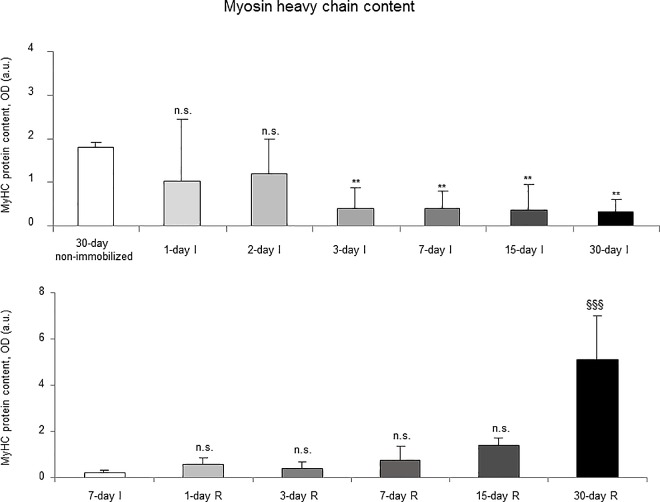
Myosin heavy chain content of all the groups in both study cohorts. Mean values and standard deviation of MyHC protein content in the gastrocnemius of the immobilization (top panel) and recovery (bottom panel) cohorts of mice, as measured by optical densities in arbitrary units (OD, a.u.). Definition of abbreviations: MyHC, Myosin Heavy Chain; OD, optical densities; a.u., arbitrary units; I, immobilization; R, recovery. Statistical significance is represented as follows: **, p≤0.01, and n.s., non-significant differences between any of the immobilized animals and the non-immobilized controls; §§§, p≤0.001, and n.s., non-significant differences between any of the R groups of animals and the 7-day I mice. Note that as arbitrary units were used for the measurement of the optical densities in each set of immunoblots (see methods), scale bars in the ordinate axes differ in the graphs of the immobilization groups from those of the recovery cohorts.

**Fig 18:**
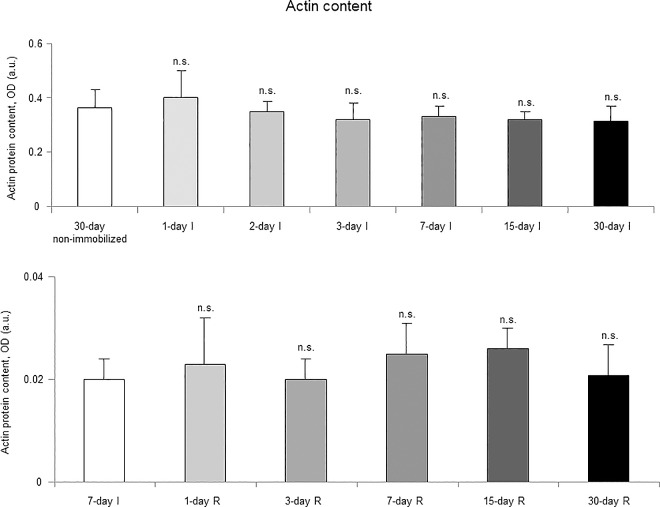
Actin content of all the groups in both study cohorts. Mean values and standard deviation of actin protein content in the gastrocnemius of the immobilization (top panel) and recovery (bottom panel) cohorts of mice, as measured by optical densities in arbitrary units (OD, a.u.). Definition of abbreviations: OD, optical densities; a.u., arbitrary units; I, immobilization; R, recovery. Statistical significance is represented as follows: n.s., non-significant differences between any of the immobilized animals and the non-immobilized controls; n.s., non-significant differences between any of the R groups of animals and the 7-day I mice. Note that as arbitrary units were used for the measurement of the optical densities in each set of immunoblots (see methods), scale bars in the ordinate axes differ in the graphs of the immobilization groups from those of the recovery cohorts.

Protein levels of contractile MyHC were significantly reduced in the gastrocnemius of 3-, 7-, 15-, and 30-day study groups of the I cohorts compared to controls (non-immobilized rodents, Fig 17, top panel, and Figure E in S1 File). Muscle actin protein levels did not significantly differ among the study groups in the I cohorts of mice (Fig 18, top panel, and Figure E in S1 File).

#### Recovery

Protein levels of MyHC were significantly increased only in the 30-day group of the R cohort compared to the controls (7-day I mice, Fig 17, bottom panel, and Figure F in S1 File). Protein levels of actin did not significantly differ among the study groups in the R cohorts of mice (Fig 18, bottom panel, and Figure F in S1 File).

### Systemic damage

#### Immobilization

**Fig 19:**
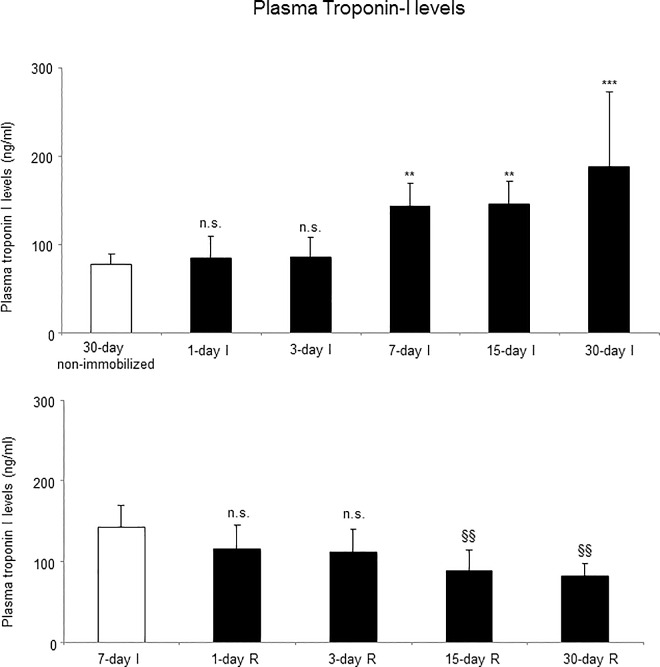
Plasma Troponin-I levels in both study cohorts. Mean values and standard deviation of fast troponin I plasma levels (ng/ml) of the immobilization (top panel) and recovery (bottom panel) cohorts of mice. Definition of abbreviations: ng, nanogram; ml, milliliter; I, immobilization; R, recovery. Statistical significance is represented as follows: **, p≤0.01, ***, p≤0.001, and n.s., non-significant differences between any of the immobilized animals and the non-immobilized controls; §§, p≤0.01, and n.s., non-significant differences between any of the R groups of animals and the 7-day I mice.

Plasma levels of fast isoform of troponin-I were significantly increased in 7-, 15-, and 30-day I study groups compared to the controls (non-immobilized mice, Fig 19, top panel).

#### Recovery

Plasma levels of fast isoform of troponin-I were significantly reduced in 15- and 30-day R study cohorts compared to the controls (7-day I mice, Fig 19, bottom panel).

### Muscle anabolism

#### Immobilization

**Fig 20:**
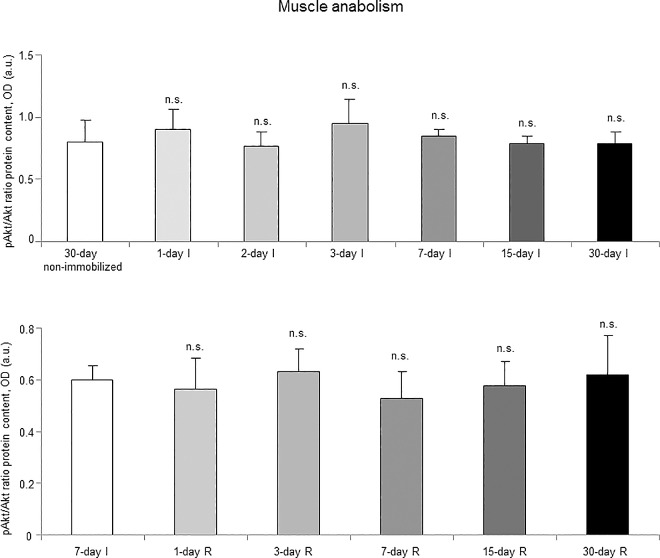
Activated Akt content of all the groups in both study cohorts. Mean values and standard deviation from the p-Akt/Akt ratio is depicted in the gastrocnemius of the immobilization (top panel) and recovery (bottom panel) cohorts of mice, as measured by optical densities in arbitrary units (OD, a.u.). Definition of abbreviations: Akt, RAC-alpha serine/threonine-protein kinase; p-Akt, phosphorylated-Akt; OD, optical densities; a.u., arbitrary units; I, immobilization; R, recovery. Statistical significance is represented as follows: n.s., non-significant differences between any of the immobilized animals and the non-immobilized controls; n.s., non-significant differences between any of the R groups of animals and the 7-day I mice. Note that as arbitrary units were used for the measurement of the optical densities in each set of immunoblots (see methods), scale bars in the ordinate axes differ in the graphs of the immobilization groups from those of the recovery cohorts.

**Fig 21:**
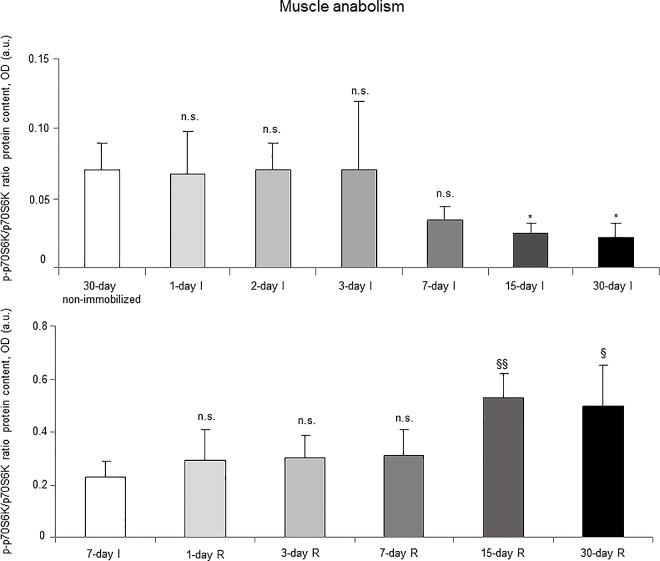
Activated p70S6K content of all the groups in both study cohorts. Mean values and standard deviation from the p-p70S6K/p70S6K ratio is depicted in the gastrocnemius of the immobilization (top panel) and recovery (bottom panel) cohorts of mice, as measured by optical densities in arbitrary units (OD, a.u.). Definition of abbreviations: p70S6K, p70 S6 kinase; p-70S6K, phosphorylated p70S6K; OD, optical densities; a.u., arbitrary units; I, immobilization; R, recovery. Statistical significance is represented as follows: *, p≤0.05, and n.s., non-significant differences between any of the immobilized animals and the non-immobilized controls; §, p≤0.05, §§, p≤0.01, and n.s., non-significant differences between any of the R groups of animals and the 7-day I mice. Note that as arbitrary units were used for the measurement of the optical densities in each set of immunoblots (see methods), scale bars in the ordinate axes differ in the graphs of the immobilization groups from those of the recovery cohorts.

**Fig 22:**
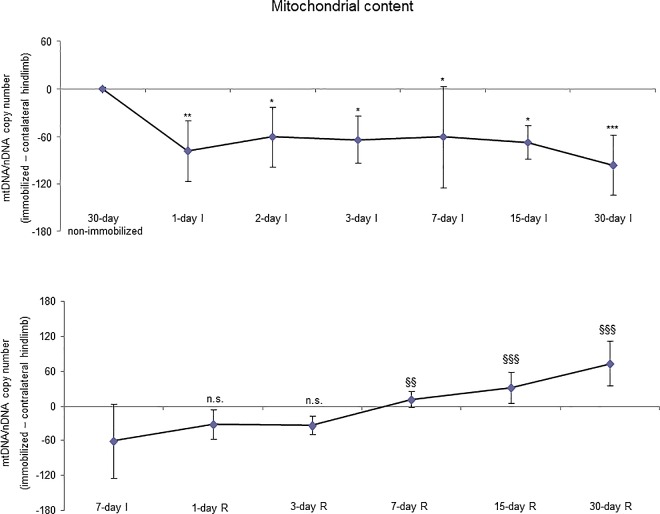
Mitochondrial content of all the groups in both study cohorts. Mean values and standard deviation of the mitochondrial DNA copy number in the gastrocnemius of the immobilization (top panel) and recovery (bottom panel) cohorts of rodents, as measured by the mtDNA/mDNA ratio in arbitrary units (a.u.). Definition of abbreviations: mt, mitochondrial; n, nuclear; DNA, deoxyribonucleic acid; I, immobilization; R, recovery. Statistical significance is represented as follows: *, p≤0.05, **, p≤0.01, and ***, p≤0.001 between any of the immobilized animals and the non-immobilized controls; §§, p≤0.01, §§§, p≤0.001, and n.s., non-significant differences between any of the R groups of animals and the 7-day I mice.

Akt protein levels (total and activated) did not significantly differ among the I cohorts (Fig 20, top panel, and Figure G in S1 File). Activated p70S6K levels significantly decreased in the gastrocnemius of 15- and 30-day study groups in the I cohorts of mice compared to the controls (non-immobilized mice, Fig 21, top panel, and Figure G in S1 File). The ratio of mtDNA/nDNA significantly deceased in the gastrocnemius of all study groups in the I cohorts of mice compared to the controls (non-immobilized mice, Fig 22, top panel).

#### Recovery

Akt protein levels (total and activated) did not significantly differ among the study groups (R cohorts, Fig 20, bottom panel, and Figure H in S1 File). Activated p70S6K levels increased in the gastrocnemius of 15- and 30-day study groups in the R cohorts compared to the controls (7-day I mice, Fig 21, bottom panel, and Figure H in S1 File). Mitochondrial content, as measured by mtDNA/nDNA, significantly increased in the gastrocnemius of 7-, 15-, and 30-day study groups in the R cohorts compared to the controls (7-day I mice, Fig 22, bottom panel).

## Discussion

The current results confirm the study hypothesis. The kinetics of relevant mechanisms shown to be involved in muscle mass loss have been described in a limb muscle of an experimental model of disuse muscle atrophy in which mice were exposed to different lengths (early and late time-points) of hindlimb immobilization. Furthermore, the kinetics of the same biological events were also analyzed in the gastrocnemius muscle during different lengths of recovery following a standardized period of hindlimb immobilization (seven days). The most interesting findings encountered in the study are discussed below.

### Time-course of muscle phenotype during immobilization and recovery

As expected, the weight of the whole gastrocnemius was significantly reduced as early as 3 days following hindlimb exposure. This is a very interesting result that is in line with those published in a recent investigation in which muscle mass loss and volume were continuously monitored using ultrasonography for two weeks in gastrocnemius and soleus of mice exposed to hindlimb unloading [39]. Nevertheless, gastrocnemius weight recovered only after 15 days of reloading following the seven-day immobilization period. As also shown in previous investigations [40,41], the sizes of both slow- and fast twitch muscle fibers were significantly smaller after seven days of immobilization than those observed in the 30-day non-immobilized control mice. Importantly, in both muscle fiber types, cross-sectional areas were similar to the control gastrocnemius following 15 days of reloading. These findings are consistent with the recovery observed in whole muscle weight at the same time-point. Thus, in this experimental model, muscle mass probably relies on the size of its muscle fibers to a great extent.

Importantly, a tendency to a slow-to-fast fiber type switch was seen in muscles of the early time-points, e.g. three- and seven-day I cohorts, despite that the statistical significance was only reached in the gastrocnemius of the 15-day I group. In these muscles, the proportions of slow-twitch muscle fibers were significantly lower, while those of fast-twitch fibers were greater. Additionally, the content of MyHC also significantly decreased in the mouse gastrocnemius of the early time-cohorts (three-day I) and thereafter. These are relevant findings, as a switch to a less fatigue-resistant phenotype has been consistently demonstrated in skeletal muscles of the lower limbs in patients with several chronic conditions such as COPD [3,11,12,34], chronic heart failure [42,43], and cancer cachexia [11]. On this basis, our data suggests that inactivity and deconditioning are likely the most significant contributors to muscle fiber atrophy and slow-to-fast fiber type switch in skeletal muscles of patients bearing chronic conditions that limit their exercise performance [3,6]. Interestingly, the switch to a less fatigue-resistant phenotype did not significantly improve during the recovery period even after 30 days of reloading. Explanations to account for this finding could be the lack of a statistically significant switch towards a less fatigue-resistant phenotype in the 7-day I cohort of mice, which was used as the control group in this set of experiments. Moreover, it is also likely that the mechanisms underlying the recovery of muscle structure following unloading may require a period longer than 30 days to fully repair the atrophying muscles. Indeed, MyHC content only significantly improved after 30 days of recovery in the mice. In fact, a minimum of eight weeks has been established for exercise training programs to target muscle structure and improve muscle metabolism [3].

In the gastrocnemius, muscle structural features such as inflammatory cell and internal nuclei counts were significantly increased as early as 3 days of unloading, especially the latter marker. Importantly, a significant reduction in total structural abnormalities and inflammatory cell counts, but not those of internal nuclei, were achieved at 3 days following reloading of the hindlimb muscles. These are interesting findings implying that the process of muscle regeneration is probably not complete even after 30 days of reloading as the number of internal nuclei remained abnormally high at all time-points in all R cohorts of mice. These features are consistent with previous results [44] in which internal nuclei were still present in the soleus of rats exposed to 1, 2 and 5 weeks of hindlimb reloading following unloading, suggesting that these fibers were still in the process of repair and/or regeneration.

Interestingly, the decrease in gastrocnemius weight, limb strength and MyHC protein content, and the increase in muscle structural abnormalities (internal nuclei) were significantly evident in animals exposed to early time-points (one- and three-day cohorts) of hindlimb immobilization. These are relevant findings suggesting that certain structural abnormalities (internal nuclei) and the physiological properties of the hindlimb muscles (strength) are particularly sensitive to experience alterations even at early stages of muscle unloading. It is likely that electrochemical properties of the muscle fibers are more prone to suffer alterations very early (1-day time cohort) following hindlimb immobilization. Specific in vitro contractility studies will help elucidate whether those factors may account for the significant reduction seen in muscle force generation in the 1-day cohort of mice, in which no signs of atrophy were detected in the gastrocnemius fibers (significant signs of muscle atrophy were indeed observed in the 7-day time-cohort). Those studies were beyond the scope of the current investigation but will certainly be a matter of research in future investigations.

Improvements in gastrocnemius weight and MyHC were observed in the late time-points (15- and 30-day R cohorts, respectively) of muscle reloading in the animals. Limb strength, slow- and fast-twitch cross-sectional areas, and total muscle structural abnormalities (inflammatory cells) significantly improved in the limb muscle of the early R cohorts. These results point to the concept that muscle regeneration, probably beyond one month, needs to be fully achieved in order for contractile proteins and gastrocnemius weight to recover to normal levels (non-exposed controls). Eventually, blood troponin I levels significantly declined in mice of the 15- and 30-day recovery periods compared to 7-day immobilization cohort. These are interesting findings which lead to the conclusion that muscle damage as represented by the presence of contractile proteins into the bloodstream starts to recover as late as 15 days after muscle reloading in the animals.

### Time-course of muscle proteolysis during immobilization and recovery

Muscle proteolysis as measured by tyrosine release assay significantly increased in the gastrocnemius of mice exposed to hindlimb immobilization for seven days and in those of the 15- and 30-day I cohorts. Additionally, expression levels of proteolytic markers such as the proteasome trypsin and chymotrypsin-like activities, 20S proteasome C8 subunit, atrogin-1, and total protein ubiquitination were also significantly greater in the muscles of the seven-day I cohort and thereafter. Nonetheless, muscle levels of the E3 ligase TRIM32 or the growth factor GDF-15 did not significantly differ among the study groups. Furthermore, levels of MURF-1 were also significantly higher in the gastrocnemius of animals from all the time-cohorts exposed to immobilization. Importantly, loss of MyHC content and atrophy of both slow- and fast-twitch muscle fibers were also seen in mice of the three-, seven-, 15-, and 30-day I cohorts, respectively. Collectively, these results suggest that the ubiquitin-proteasome system and the E3 ligases MURF-1 and atrogin-1 may underlie muscle mass and protein loss in this mouse model of disuse muscle atrophy in the fast-twitch gastrocnemius. These findings are in line with previous investigations conducted on other experimental models of disuse muscle atrophy in rodents [40,45]. Differences in the duration and/or intensity of the unloading exposure may account for potential discrepancies in the levels and time-course expression of the proteolytic events involved in muscle mass loss among the different models.

A significant novel result in the investigation was the rise in muscle troponin I levels detected in the blood of mice of the relatively late time-cohorts: seven-, 15-, and 30-day I groups. Troponin I binds to actin in thin myofilaments in order to maintain the actin-tropomyosin in place within the skeletal muscle fibers. Detection of troponin I in the bloodstream of patients [30], healthy subjects [28,29], and animal models [31] is a surrogate of muscle damage. In the investigation, the fast troponin I isoform was specifically detected in the blood samples of all study groups of animals. These findings imply that extensive damage must have taken place in the mouse gastrocnemius muscles in response to hindlimb unloading, especially in those animals exposed to immobilization for relatively longer periods of time.

Relevant findings in the investigation were also the significant decline in mitochondrial content in the early (one-day I) and late time-cohorts. In fact, it has been demonstrated that alterations in mitochondrial structural and functional integrity are critical in the initiation of muscle protein degradation following disuse muscle atrophy [46–48]. Mitochondrial E3 ubiquitin protein ligase-1 was upregulated by forkhead box (Fox)O pathway inducing mitophagy in models of muscle wasting [46]. In the current study, levels of E3 ligase MURF-1 were also significantly reduced in the one-day I time-cohort of mice and thereafter. Recently, mitophagy via increased FoxO1 activity was also demonstrated in the limb muscles of mice exposed to hindlimb immobilization for several time-points [49,50]. Taken together, these findings suggest that loss of mitochondria may be a major trigger of enhanced protein breakdown in models of disuse muscle atrophy. Reloading significantly attenuated the decline in mitochondrial content in mouse muscles of the seven-day R cohort and thereafter. Signaling mechanisms that modulate mitophagy are likely to mediate the restoration of mitochondrial content during muscle reloading. Nonetheless, identification of the signaling pathways potentially involved in this process will be the matter of future research.

As early as one day after muscle reloading (recovery period), tyrosine release levels were significantly decreased in the mouse gastrocnemius, and levels remained low thereafter in all the study cohorts. Moreover, trypsin- and chymotrypsin-like activities and MURF-1 were also significantly reduced in the study muscle after three days of hindlimb reloading and remained low in the all the R cohorts of animals. Additionally, expression levels of 20S proteasome C8 subunit, total protein ubiquitination, and atrogin-1 were also significantly attenuated in the gastrocnemius after seven days of reloading and thereafter in the R cohorts compared to those in the 7-day immobilized controls. Besides, the cross-sectional areas of both type I and type II fibers were also significantly larger after seven days of hindlimb reloading and thereafter in the R cohorts of mice. Similar findings were also reported for the gastrocnemius in myostatin deficient mice exposed to muscle unloading and reloading (weight-bearing model) of the hindlimb [51]. Collectively, these findings suggest that the time-course of molecular and cellular events follows a very consistent pattern during muscle reloading following disuse muscle atrophy. The advantage of the current model is that it faithfully represents what happens in actual clinical settings after a period of bed rest, in which patients are forced to initiate their daily life activities.

## Conclusions

The current non-invasive mouse model of disuse limb muscle atrophy followed a very specific and consistent program of molecular and cellular events that led to muscle mass loss and reduced strength, enhanced proteolysis via ubiquitin-proteasome pathway, injury (muscle structure and troponin I release), and decreased both mitochondrial content and MyHC in the gastrocnemius. Unloading of the muscle following removal of the splint significantly improved the alterations seen during unloading, characterized by a specific kinetic profile of molecular events involved in muscle regeneration. These findings have implications in patients with chronic diseases including cancer in whom physical activity may be severely compromised.

## Supporting Information

S1 File Detailed methodologies. Figure A. Figure B. Figure C. Figure D. Figure E. Figure F. Figure G. Figure H. Table A.
(DOCX)

S1 Video Mouse physical activity in the cage. (AVI)

S2 Video Grip strength maneuver in mice. (AVI)
