# Supplementary material for: Time-Course of Muscle Mass Loss, Damage, and Proteolysis in Gastrocnemius following Unloading and Reloading: Implications in Chronic Diseases
Source: PLoS One. 2016 Oct 28;11(10):e0164951. doi: 10.1371/journal.pone.0164951 (PMC5085049; doi:10.1371/journal.pone.0164951)
Supplement: S1 File — Figure A. Figure B. Figure C. Figure D. Figure E. Figure F. Figure G. Figure H. Table A. (DOCX) [file pone.0164951.s001.docx]

**Supporting Information File S1**

**time-course OF MUSCLE MASS LOSS, DAMAGE, and PROTEOLYSIS in gastrocnemius following unloading and reloading: implications in chronic diseaseS**

**Alba Chacon-Cabrera,** **Elena Lund-Palau, Joaquim Gea, Esther Barreiro**

**MATERIALS AND METHODS**

**Animal experiments**

Female C57BL/6J mice (10 weeks old, weight ~20 g) were obtained from Harlan *Interfauna Ibérica SL* (Barcelona, Spain). Mice were kept under pathogen-free conditions in the animal house facility at Barcelona Biomedical Research Park (PRBB), with a 12:12 h light: dark cycle.

Mice were exposed to unilateral hindlimb immobilization as previously described to reproduce a model of disuse muscle atrophy [1]. Briefly, the left hindlimb was shaved with clippers and was enveloped using surgical tape. The hindlimb was introduced in a 1.5 mL microcentrifuge tube with cover and bottom lids removed, while maintaining the foot in a plantar-flexed position to induce the maximal atrophy of the target limb muscle. The entire procedure was carried out using mouse restrainers and without anesthesia. As the weight of the tube was approximately 0.6 g, it did not interfere with the usual mobility of the mice. In the study, the following control groups of mice were used for different purposes. Firstly, in order to assess potential differences in body weight and food intake, age-matched non-immobilized control mice were used for all the study groups (see below). Secondly, in order to explore potential differences in the variables muscle weight, tyrosine release, proteasome activities, mitochondrial content, muscle phenotype and morphometry, and structural abnormalities, the contralateral non-immobilized limb was used for these experiments. Thirdly, with the aim to evaluate potential differences in several markers of proteolysis, signaling pathways, and structural and functional proteins using immunoblotting, and blood troponin I levels using enzyme-linked immunoSorbent assay (ELISA) a group of 30-day non-immobilized mice (period long enough to ensure potential differences if any) and a group of 7-day immobilized rodents, were used as the control group of the immobilization and recovery time-cohorts, respectively.

As shown in Fig A in S1 File, three different approaches were taken in the investigation: 1) non-immobilization group, 2) immobilization time-cohorts (I groups), and 3) recovery time-cohorts (R groups), in which the left hindlimb of the mice was immobilized for seven consecutive days, time at which the splint was removed to let the animals move freely in their cages to evaluate muscle recovery at different time-points. Afterwards, animals were randomly assigned to the following groups (N=10/group): 1) 30-day non-immobilized control group; 2) mice immobilized for one day (1-day I); 3) mice immobilized for two days (2-day I); 4) mice immobilized for three days (3-day I); 5) mice immobilized for seven days (7-day I); 6) mice immobilized for fifteen days (15-day I); 7) mice immobilized for thirty days (30-day I); 8) mice exposed to seven days of unilateral hindlimb immobilization followed by one day recovery (1-day R); 9) mice exposed to seven days of unilateral hindlimb immobilization followed by three days recovery (3-day R); 10) mice exposed to seven days of unilateral hindlimb immobilization followed by seven days recovery (7-day R); 11) mice exposed to seven days of unilateral hindlimb immobilization followed by fifteen days recovery (15-day R); 12) mice exposed to seven days of unilateral hindlimb immobilization followed by thirty days recovery (30-day R).

All animal experiments were conducted in the animal facilities at *Parc de Recerca Biomèdica de Barcelona* (PRBB). This controlled study was designed in accordance with the ethical standards on animal experimentation (EU 2010/63 CEE, *Real Decreto* 53/2013 BOE 34, Spain) at PRBB and the Helsinki convention for the use and care of animals. Ethical approval was obtained by the Animal Research Committee (Animal welfare department in Catalonia, EBP-13-1485).

***In vivo* measurements in the mice**

In all the study animals, body weight and food intake were measured at every time-point, and food and water were supplied ad libitum for the entire duration of the immobilization or recovery periods. In all mice, limb strength was determined on day 0, day 30 (non-immobilized controls), and right at the end of each immobilization or recovery time-points (as describe above) using a grip strength meter (Bioseb, Vitrolles Cedex, France) following previously published methodologies, in which grip strength was also the end-point parameter in the different experimental models [2–5]. As shown in Fig B in S1 File, and in S2 video, grip strength was assessed in the four limbs at the same time in all mice. In all the animals, limb strength gain was calculated as the percentage of the measurements performed at the end of the study period with respect to the same measurements obtained at baseline (grip strength at the end of the study period–grip strength on day 0)/ grip strength on day 0 x 100).

**Sacrifice and sample collection**

Mice from all the experimental groups were sacrificed after the corresponding immobilization or recovery time-cohorts, or after 30 days (non-immobilized control group). Each mouse was previously inoculated intraperitoneally with 0.1 mL sodium pentobarbital (60 mg/Kg). In all cases, the pedal and blink reflexes were evaluated in order to verify total anesthetic depth. The following samples were obtained from all the animals at the time of sacrifice: blood and gastrocnemius muscle. Sacrifice of animals was carried out through diaphragm extraction. The absence of the diaphragm entailed an immediate cardiac arrest and resulted in animal death. Blood samples, which were obtained through puncture of the saphenous vein, were centrifuged at 1,200 rpm for 15 minutes to yield plasma. Muscle samples were snap-frozen in liquid nitrogen to be thereafter stored frozen at -80ºC to be further used for the molecular analyses. Moreover, another fragment of the muscle specimens was paraffin-embedded to be used for the assessment of muscle structure abnormalities and fiber type composition and morphometry.

**Biological analyses**

*ELISA plasma skeletal muscle troponin-I levels.* Skeletal muscle troponin-I levels were quantified in plasma of the following groups of animals: 1-day I, 7-day I, 15-day I, 30-day I, 1-day R, 15-day R, 30-day R, and the 30-day non-immobilized controls using a specific sandwich ELISA kit (Life Diagnostics Inc., West Chester, PA, USA) as previously shown [6–9]. Before the start of the assay, samples and reagents were equilibrated to room temperature. A standard curve was always run with each assay run. Standards (100 microL) and the protocol were performed as indicated by the manufacturer’s instructions. All reagents used in these experiments were part of the specific ELISA kit. For all the study samples equal volume (100 microL total volume) of diluted plasma (1:3 dilution) were always loaded in duplicates onto the pre-coated ELISA-plate wells. Samples were incubated with 100 microL horseradish peroxidase (HRP)-secondary antibody on an orbital micro-plate shaker at 150 rpm and 25ºC for one hour. The wells were then washed six times with the wash solution and incubated with 100 microL tetramethylbenzidine (TMB) reagent on an orbital micro-plate shaker at 150 rpm at 25ºC for 20 minutes. Finally, the enzyme reaction was stopped by adding 100 microL stop solution to the wells. Absorbances were read in a microplate reader at 450 nm using as a reference filter that of 655 nm. Intra-assay coefficients of variation for the plasma skeletal muscle troponin-I levels ranged from 2% to 10%. As all the samples were analyzed on the same day, no inter-assay coefficients of variation could be calculated.

*Muscle DNA isolation***.** Total DNA, including mitochondrial and nuclear DNA was isolated from gastrocnemius muscle of all mouse experimental groups using QIAmp DNA Mini Kit (QiAgen, GmbH, Germany), following the manufacturer’s protocol of DNA purification from tissues, and without the use of RNase A, as previously shown [10]. Total DNA obtained from muscles was quantified using a spectrophotometer (Thermo Scientific, Waltham, MA, USA).

*Absolute quantification by real-time PCR of DNAs.* Mitochondrial DNA (mtDNA) copy numbers were estimated through the quantification of the mtDNA to nuclear DNA (nDNA) ratio (mtDNA/nDNA). The content of mtDNA was determined using singleplex (amplifying one target sequence per well) qRT-PCR analyses, and corrected by the simultaneous measurements of a single copy of nuclear angiogenin-1 (ANG1) gene. The mtDNA 16S rRNA primers and TaqMan probe were used as also previously reported [11]. The primers for absolute quantification of qRT-PCR assessment of mtDNA were: 16S Fw (5’AATGGTTCGTTTGTTCAACGATT3’) and 16S Rv (5’AGAAACCGACCTGGATTGCTC3’). The TaqMan probe (FAM-5’AAGTCCTACGTGATCTGAGTT3’-MGB) was labeled at the 5’ end with the fluorescent reporter FAM (6-carboxy fluorescein). A commercial assay was used in order to quantify the nuclear single copy gene ANG1 (Mm00833184_s1, Applied Biosystems) [11]. The nDNA PCR reactions contained 1X TaqMan Universal PCR Master Mix (Applied Biosystems, 4440040), 1 microL ANG1 assay, and 10 ng of total DNA in a total volume of 10 microL. The 10- microL PCR reactions for the quantification of mtDNA contained 1X TaqMan Universal PCR Master Mix (Applied Biosystems, 4440040), 300 nM of each mtDNA primer, 150 nM of mtDNA TaqMan probe, and 10 ng of total DNA. In these experiments, PCR conditions were established following previously published methodologies [10,11]. PCR calibration curves were set separately for mtDNA and nDNA copy numbers to estimate the copy numbers of each type of DNA. The ratio of mtDNA to nDNA was further calculated and corresponding data were expressed as mtDNA/nDNA for the gastrocnemius in all study groups [11]. These results were used as a measure of mitochondrial content in the muscles.

*Immunoblotting of 1D electrophoresis.* Protein levels of the different molecular markers analyzed in the study were explored by means of immunoblotting procedures as previously described [3–5]. Briefly, frozen muscle samples from the gastrocnemius muscle of all mouse experimental groups were homogenized in a buffer containing 50 mM 4-(2-hydroxyethyl)-1-piperazineethanesulfonic acid (HEPES), 150 mM NaCl, 100 mM NaF, 10 mM Na pyrophosphate, 5 mM ethylenediaminetetraacetic acid (EDTA), 0.5% Triton-X, 2 micrograms/mL leupeptin, 100 micrograms/mL phenylmethanesulfonyl fluoride (PMSF), 2 micrograms/mL aprotinin and 10 micrograms/mL pepstatin A. Moreover, myofibrillar proteins were also isolated in order to identify levels of actin and myosin heavy chain (MyHC) as previously reported [3,4,12,13]. The entire procedures were always conducted at 4ºC. Protein levels in crude homogenates were spectrophotometrically determined with the Bradford method [14] using triplicates in each case and bovine serum albumin (BSA) as the standard (Bio-Rad protein reagent, Bio-Rad Inc., Hercules, CA, USA). The final protein concentration in each sample was calculated from at least two Bradford measurements that were almost identical. Equal amounts of total protein (ranging from 5 to 200 micrograms, depending on the antigen and antibody) from crude muscle homogenates were always loaded onto the gels, as well as identical sample volumes/lanes. For the purpose of comparisons among the different groups of experimental and control rodents, muscle sample specimens were always run together and kept in the same order. Two independent sets of immunoblots were conducted, in which immobilized groups and recovery groups were run separately. Four fresh 10-well mini-gels were always simultaneously loaded for each of the antigens. Experiments were confirmed twice for all the antigens analyzed in the investigation. Fresh gels were specifically loaded for each of the antigens in muscle specimens of all mice in most of cases. However, in a few cases, antigens were identified from stripped membranes.

Proteins were then separated by electrophoresis, transferred to polyvinylidene difluoride (PVDF) membranes, blocked with bovine serum albumin and incubated overnight with selective primary antibodies. Protein levels of signaling pathways, proteolysis, anabolism and muscle contractile proteins were identified in the gastrocnemius using specific primary antibodies: α-actin (anti-α-sarcomeric actin antibody, clone 5C5, Sigma-Aldrich), myosin heavy chain (anti-MyHC antibody, clone A4.1025, Upstate-Millipore), RAC-alpha serine/threonine-protein kinase (Akt) and p-Akt (anti-Akt and anti-p-Akt antibodies, Cell Signaling Technology), p70 S6 kinase (p70S6K) and p-p70S6K (anti- p70S6K and anti-p-p70S6K antibodies, Cell Signaling Technology), total ubiquitinated proteins (anti-ubiquitinated proteins antibody, Boston Biochem), 20S proteasome subunit C8 (anti-C8 antibody, Biomol), tripartite motif containing 32 (TRIM32) (anti-TRIM32 antibody, Santa Cruz Biotechnology), ubiquitin-ligase atrogin-1 (anti-atrogin-1 antibody, Santa Cruz Biotechnology), ubiquitin-ligase muscle ring finger (MURF)-1 (anti-MURF-1 antibody, Everest Biotech), growth differentiation factor 15 (GDF-15) (anti-GDF15 antibody, Santa Cruz Biotechnology) and glyceraldehyde-3-phosphate dehydrogenase (GAPDH) (anti-GAPDH antibody, Santa Cruz Biotechnology). Antigens from all samples were detected with horseradish peroxidase (HRP)-conjugated secondary antibodies and a chemiluminescence kit. For each of the antigens, samples from the different groups were always detected in the same picture under identical exposure times. The specificity of the different antibodies was confirmed by omission of the primary antibody, and incubation of the membranes only with secondary antibodies.

PVDF membranes were scanned with the Molecular Imager Chemidoc XRS System (Bio–Rad Laboratories, Hercules, CA, USA) using the software Quantity One version 4.6.5 (Bio–Rad Laboratories). Optical densities of specific proteins were quantified using the software Image Lab version 2.0.1 (Bio-Rad Laboratories). Final optical densities obtained in each specific group of subjects and muscle corresponded to the mean values of the different samples (lanes) of each of the antigens studied. In order to validate equal protein loading among various lanes, the glycolytic enzyme GAPDH was used as the protein loading controls in all the immunoblots.

Standard stripping methodologies were employed when detection of the antigens required the loading of a relatively greater amount of total muscle protein. Membranes were stripped of primary and secondary antibodies after a 30-minute wash with a specific stripping solution [25 mM glycine, pH 2.0 and 1% sodium dodecyl sulfate (SDS)] followed by two consecutive 10-minute washes containing phosphate buffered saline with tween (PBST) at room temperature. Membranes were blocked with bovine serum albumin (BSA) and reincubated with primary and secondary antibodies following the procedures described above.

*Protein catabolism*. Protein degradation in muscles was explored on the basis of the rate of production of free tyrosine from tissue proteins as previously described [3,4,15,16]. As muscles cannot synthesize or degrade this amino acid, its accumulation reflects the net degradation of proteins. All incubations were performed at 35ºC in a 95% air-5% CO_2_ mixture. Briefly, whole excised muscles were placed in individual tissue chambers containing 3 mL of TKH1 buffer (127.8 mM NaCl, 4.7 mM KCl, 2.4 mM MgSO_4_·7H_2_O, 1.2 mM KH_2_PO_4_, 2.5 mM CaCl_2_·2H_2_O, 20 mM Hepes, 170 microM L-leucine, 100 microM L-isoleucione, 200 microM L-valine and 0.5 M glucose) and were preincubated for 15 minutes. After the preincubation, the buffer was extracted and 3 mL of TKH2 (TKH1 supplemented with 500 mM cicloheximide) buffer were added to the same chamber, and samples were then incubated for 15 minutes. Thereafter, the buffer was extracted and replaced with 4 mL of fresh TKH2, and samples were then incubated for 2 hours. Immediately afterwards, the buffer was recovered and stored at -20ºC up until the tyrosine release measurements were performed following these procedures: 1.4 mL sample, blank (127.8 mM NaCl, 4.7 mM KCl, 2.4 mM MgSO_4_·7H_2_O, 1.2 mM KH_2_PO_4_, 2.5 mM CaCl_2_·2H_2_O and 20 mM HEPES) or standards (tyrosine in blank from 0 to 2.5 micrograms/mL) were combined with 250 microL of 30% trichloroacetic acid (TCA) in a centrifuge tube and centrifuged at 2,500 rpm for 15 minutes, supernatant was recovered and placed on a new tube. Supernatant was combined with 300 microL of 1-nitroso-2-naphthol 0.1% in 95% ethanol and 300 microL of nitric acid mixture, and incubated for 30 minutes at 55ºC and then 15 more minutes at room temperature. Four mL of ethylene dichloride were added to the tubes and were then shaken. The tubes were then centrifuged at 1,500 rpm for 4 minutes. Four-hundred microL of supernatant were transferred to a 96-well black microplate and measurements of tyrosine fluorescence was performed at 570 nm, resulting from its activation at 460 nm using a fluorometer (Infinite M200, TECAN, Männedorf, Switzerland). The results were expressed as nmol of tyrosine/mg of muscle/2 hours of incubation.

*Proteasome activities.* The first step included the isolation of the proteasome, in which previously published procedures were followed [3,17]. Approximately 100 mg of frozen muscle samples from the gastrocnemius of all groups of mice were homogenized using a polytron homogenizer and 6 volumes (w/v) of homogenization buffer [50 mM Tris HCl, 5 mM MgCl_2_, 250 mM sucrose, 1 mM dithiothreitol (DTT), 0.2 mM PMSF, and a cocktail of proteases inhibitors (2 micrograms/mL leupeptin, 100 micrograms/mL PMSF, 2 micrograms/mL aprotinin, and 10 micrograms/mL pepstatin A), pH=7.5]. The entire procedures were always conducted at 4ºC. Subsequently, crude homogenates were centrifuged at 10,000 g at 4ºC for 20 minutes. Afterwards, pellets were discarded and supernatants were transferred into a new tube and centrifuged again at 100,000 g at 4ºC for one hour. Again, supernatants were collected and centrifuged at 100,000 g at 4ºC for five hours. Finally, supernatants were discarded and pellets were re-suspended with 150 microL of a medium buffer containing 50 mM Tris-HCl, 5 mM MgCl_2_, and 20% glycerol, at a pH=7.5. Protein levels in crude homogenates were spectrophotometrically determined with the Bradford method [14]. In order to verify the correct proteasome isolation an immunoblotting against alpha7 subunit 20S proteasome (clone MCP72; Biomol, Plymouth Meeting, PA, USA) were performed in all samples.

*Chymotrypsin-like activity.* In the gastrocnemius muscle of all study animals, chymotrypsin-like activity was evaluated according to the cleavage of the fluorescent substrate Suc-LLVY-AMC (Biomol International, Plymouth Meeting, PA, EEUU) and the generation of a fluorogenic product, the methyl coumarilamide (AMC) following previously published methodologies [3,17]. Briefly, 5 micrograms of protein was combined in a tube with 50 microL of assay buffer [20 mM Tris-HCl pH=7.5, 1 mM EDTA, 1 mM NaN_3_, 1 mM DTT] together with 50 microL of 200 microM LLVY-substrate assay solution. At this point, sample tubes were maintained on ice. Subsequently, a stock solution of 1 mg of AMC/100 microL of DMSO was prepared for the standards. Standard curve (AMC in blank from 0 to 16 microM) was prepared directly into a 96-well transparent microplate and loaded in duplicates with a final volume of 40 microL per well. Sample tubes and the plate with the standard curve were incubated at 37ºC for 30 minutes. Afterwards, 40 microL of each sample was loaded in duplicates onto the plate. Finally, 200 microL of stop solution was poured into each well and measurements of fluorescence were performed at 460 nm, resulting from its activation at 360 nm using a fluorometer (Infinite M200, TECAN, Männedorf, Switzerland). The results were expressed as nmol of chymotrypsin-like activity/mg of muscle protein. Intra-assay coefficients of variation ranged from 1.3% and 16.22%.

*Trypsin-like activity.* In gastrocnemius muscles of all study animals, trypsin-like activity was evaluated according to the cleavage of the fluorescent substrate Boc-Leu-Arg-Arg-AMC (Biomol International, Plymouth Meeting, PA, EEUU) and the generation of a fluorogenic product, the methyl coumarilamide (AMC) following other published methodologies [3,17]. Briefly, five micrograms of protein was combined in a tube with 50 microL of assay buffer [20 mM Tris-HCl pH=7.5, 1 mM EDTA, 1 mM NaN_3_, 1 mM DTT] together with 50 microL of 200 microM Boc-substrate assay solution. At this point, sample tubes were maintained on ice. Subsequently, a stock solution of 1 mg of AMC/100 microL of DMSO was prepared for the standards. Standard curve (AMC in blank from 0 to 16 microM) was prepared directly into a 96-well transparent microplate and loaded in duplicates with a final volume of 40 microL per well. Sample tubes and the plate with the standard curve were incubated at 37ºC for 30 minutes. Afterwards, 40 microL of each sample was loaded in duplicates onto the plate. Finally, 200 microL of Stop solution was poured into each well and measurements of fluorescence were performed at 460 nm, resulting from its activation at 360 nm using a fluorometer (Infinite M200, TECAN, Männedorf, Switzerland). The results were expressed as nmol of Trypsin-like activity/mg of muscle protein. Intra-assay coefficients of variation ranged from 1.2% and 16.47%.

*Immunohistochemistry.* On 3-micrometer muscle paraffin-embedded sections from gastrocnemius muscle of all study groups, MyHC-I and –II isoforms were identified using anti-MyHC-I (clone MHC, Biogenesis Inc.) and anti-MyHC-II antibodies (clone MY-32, Sigma-Aldrich), respectively, as published elsewhere [3–5]. The cross-sectional area, mean least diameter, and proportions of type I and type II fibers were assessed using a light microscope (Olympus, Series BX50F3, Olympus Optical Co., Hamburg, Germany) coupled with an image-digitizing camera (Pixera Studio, version 1.0.4, Pixera Corporation, Los Gatos, CA, USA) and a morphometry program (NIH Image, version 1.60, Scion Corporation, Frederick, MD, USA). At least 100 fibers were measured and counted in each type of muscle specimen from all groups of mice.

*Muscle structure abnormalities.* The area fraction of normal and abnormal muscle was evaluated on 3-micrometer paraffin-embedded sections of the gastrocnemius of all study groups muscles following previously published methodologies [3–5]. Briefly, normal and abnormal tissue was quantified using computer-assisted point counting in all the limb muscle sections, previously stained with hematoxylin-eosin. A grid of 63 point-intercepts (7 x 9 rectangular pattern), built by means of the software Imaging Cell-B (Olympus Corporation), was superimposed onto the image of the muscle cross section at a magnification of x400 under the light microscope (Olympus BX 61, Olympus Corporation) using an image digitizing camera (Olympus DP 71, Olympus Corporation). Each point-intercept was assigned to a specific category and entered into the software. Categories for point counting were defined as follows: 1) normal muscle, 2) internal nucleus, 3) inflammatory cell, 4) lipofuscin, 5) abnormal viable, 6) inflamed/necrotic, 7) vessel, and 0) no count. The area fraction for each category was defined as the percentage of points that fell on each of these traits relative to the total number of points superimposed on all viable fields (all features except for categories 0 and 7) of each cross section. The area fraction of normal muscle was equivalent to the proportions of points falling in category 1, while the area fraction of abnormal muscle was determined by calculation of the proportion of points included in the other categories (categories 2 to 6) ( Fig C in S1 File).

**Statistical Analysis**

Normality of the study variables were checked using the Shapiro-Wilk test. Physiological, structural, and molecular results are expressed as mean (standard deviation). The following statistical approaches were used in the study for different purposes. Firstly, total body weight and food intake of mice from each experimental group (I and R cohorts) were compared with their corresponding age-matched non-immobilized controls using the unpaired Student’s T-test. For each pair, a level of significance of *P*≤ 0.05 was established.

Secondly, deltas of the difference of mean values of the results obtained in the gastrocnemius of the immobilized hindlimb with respect to those of the contralateral non-immobilized hindlimb, were also calculated in each animal from both cohorts (I and R), and from 30-day non-immobilized animals for another set of comparisons for the following variables: muscle weight, tyrosine release, proteasome activities, mitochondrial content, muscle phenotype and morphometry, and structural abnormalities. In this case, results are expressed as mean delta (standard deviation). Deltas obtained from each group were compared as follows: 1) deltas from mice of the immobilized cohorts (I groups) versus deltas of 30-day non-immobilized controls and 2) deltas from mice of the recovery cohorts (R groups) versus deltas of mice that were exposed to seven days immobilization (7-day I, control group). Potential significant differences were assessed using one-way analysis of variance (ANOVA) with *Dunnett’s post hoc* analysis to adjust for multiple comparisons among the study groups. A level of significance of P≤ 0.05 was established.

Thirdly, for the following variables: 20S proteasome C8, ubiquitin, E3-ligases, GDF15, MyHC, actin, Akt, P70S6K, and troponin I, comparisons were made between each group of mice from the different time-points of the I cohorts and the 30-day non-immobilized animals, and from the different time-points of the R cohorts compared with 7-day I. The following comparisons were performed to explore potential differences among the groups: 1) mice from the immobilized cohorts (I groups) versus the 30-day non-immobilized controls and 2) animals from the recovery cohorts (R groups) versus animals immobilized for 7 days (7-day I, control group). In these comparisons, potential significant differences were assessed using one-way analysis of variance (ANOVA) with *Dunnett’s post hoc* analysis to adjust for multiple comparisons among the study groups. A level of significance of *P*≤ 0.05 was established.

The sample size chosen was based on previous studies [3–5,18], where very similar approaches were employed. In addition, statistical power was calculated using specific software (StudySize 2.0, CreoStat HB, Frolunda, Sweden). Limb strength gain and changes in myofiber cross sectional area were selected as the target variables to estimate the statistical power in the study. On the basis of a standard power statistics established at a minimum of 80% and assuming an alpha error of 0.05, the statistical power was sufficiently high to detect a minimum difference of 25 points of delta in limb strength gain and 300 points of delta in myofiber cross sectional area respectively, among the different study groups for the given sample size and standard deviations.

Reference List

1. Lang SM, Kazi AA, Hong-Brown L, Lang CH (2012) Delayed recovery of skeletal muscle mass following hindlimb immobilization in mTOR heterozygous mice. PLoS One 7: e38910. 10.1371/journal.pone.0038910 [doi];PONE-D-12-00485 [pii].

2. Barreiro E, Marin-Corral J, Sanchez F, Mielgo V, Alvarez FJ, et al. (2010) Reference values of respiratory and peripheral muscle function in rats. J Anim Physiol Anim Nutr (Berl) 94: e393-e401. JPN1027 [pii];10.1111/j.1439-0396.2010.01027.x [doi].

3. Barreiro E, Puig-Vilanova E, Marin-Corral J, Chacon-Cabrera A, Salazar-Degracia A, et al. (2015) Therapeutic Approaches in Mitochondrial Dysfunction, Proteolysis, and Structural Alterations of Diaphragm and Gastrocnemius in Rats With Chronic Heart Failure. J Cell Physiol . 10.1002/jcp.25241 [doi].

4. Chacon-Cabrera A, Fermoselle C, Urtreger AJ, Mateu-Jimenez M, Diament MJ, et al. (2014) Pharmacological strategies in lung cancer-induced cachexia: effects on muscle proteolysis, autophagy, structure, and weakness. J Cell Physiol 229: 1660-1672. 10.1002/jcp.24611 [doi].

5. Chacon-Cabrera A, Fermoselle C, Salmela I, Yelamos J, Barreiro E (2015) MicroRNA expression and protein acetylation pattern in respiratory and limb muscles of Parp-1(-/-) and Parp-2(-/-) mice with lung cancer cachexia. Biochim Biophys Acta 1850: 2530-2543. S0304-4165(15)00262-7 [pii];10.1016/j.bbagen.2015.09.020 [doi].

6. Chapman DW, Simpson JA, Iscoe S, Robins T, Nosaka K (2013) Changes in serum fast and slow skeletal troponin I concentration following maximal eccentric contractions. J Sci Med Sport 16: 82-85. S1440-2440(12)00104-1 [pii];10.1016/j.jsams.2012.05.006 [doi].

7. Foster GE, Nakano J, Sheel AW, Simpson JA, Road JD, et al. (2012) Serum skeletal troponin I following inspiratory threshold loading in healthy young and middle-aged men. Eur J Appl Physiol 112: 3547-3558. 10.1007/s00421-012-2337-5 [doi].

8. Simpson JA, Labugger R, Collier C, Brison RJ, Iscoe S, et al. (2005) Fast and slow skeletal troponin I in serum from patients with various skeletal muscle disorders: a pilot study. Clin Chem 51: 966-972. clinchem.2004.042671 [pii];10.1373/clinchem.2004.042671 [doi].

9. Vassallo JD, Janovitz EB, Wescott DM, Chadwick C, Lowe-Krentz LJ, et al. (2009) Biomarkers of drug-induced skeletal muscle injury in the rat: troponin I and myoglobin. Toxicol Sci 111: 402-412. kfp166 [pii];10.1093/toxsci/kfp166 [doi].

10. Andreu AL, Martinez R, Marti R, Garcia-Arumi E (2009) Quantification of mitochondrial DNA copy number: pre-analytical factors. Mitochondrion 9: 242-246. S1567-7249(09)00039-7 [pii];10.1016/j.mito.2009.02.006 [doi].

11. McDermott-Roe C, Ye J, Ahmed R, Sun XM, Serafin A, et al. (2011) Endonuclease G is a novel determinant of cardiac hypertrophy and mitochondrial function. Nature 478: 114-118. nature10490 [pii];10.1038/nature10490 [doi].

12. Fermoselle C, Rabinovich R, Ausin P, Puig-Vilanova E, Coronell C, et al. (2012) Does oxidative stress modulate limb muscle atrophy in severe COPD patients? Eur Respir J 40: 851-862. 09031936.00137211 [pii];10.1183/09031936.00137211 [doi].

13. Marin-Corral J, Minguella J, Ramirez-Sarmiento AL, Hussain SN, Gea J, et al. (2009) Oxidised proteins and superoxide anion production in the diaphragm of severe COPD patients. Eur Respir J 33: 1309-1319.

14. Bradford MM (1976) A rapid and sensitive method for the quantitation of microgram quantities of protein utilizing the principle of protein-dye binding. Anal Biochem 72: 248-254. S0003269776699996 [pii].

15. Furuno K, Goodman MN, Goldberg AL (1990) Role of different proteolytic systems in the degradation of muscle proteins during denervation atrophy. J Biol Chem 265: 8550-8557.

16. Tischler ME, Desautels M, Goldberg AL (1982) Does leucine, leucyl-tRNA, or some metabolite of leucine regulate protein synthesis and degradation in skeletal and cardiac muscle? J Biol Chem 257: 1613-1621.

17. van Hees HW, Li YP, Ottenheijm CA, Jin B, Pigmans CJ, et al. (2008) Proteasome inhibition improves diaphragm function in congestive heart failure rats. Am J Physiol Lung Cell Mol Physiol 294: L1260-L1268. 00035.2008 [pii];10.1152/ajplung.00035.2008 [doi].

18. Fermoselle C, Sanchez F, Barreiro E (2011) [Reduction of muscle mass mediated by myostatin in an experimental model of pulmonary emphysema]. Arch Bronconeumol 47: 590-598. S0300-2896(11)00300-0 [pii];10.1016/j.arbres.2011.07.008 [doi].

**Table A.** **Food intake in all groups of the immobilization and recovery study cohorts.**

|  | **Food intake (g/24h)** | |
| --- | --- | --- |
|  | **Non- immobilized age-matched mice** | **Immobilized mice** |
| **1-Day I** | 3.6 (0.2) | 3.4 (0.2), n.s. |
| **2-Day I** | 3.4 (0.1) | 3.5 (0.2), n.s. |
| **3-Day I** | 3.6 (0.4) | 3.4 (0.1), n.s. |
| **7-Day I** | 3.5 (0.3) | 3.4 (0.3), n.s. |
| **15-Day I** | 3.5 (0.1) | 3.4 (0.1), n.s. |
| **30-Day I** | 3.5 (0.1) | 3.4 (0.1), n.s. |
|  | **Non- immobilized age-matched mice** | **Recovery mice** |
| **1-Day R** | 3.5 (0.2) | 3.4 (0.1), n.s. |
| **3-Day R** | 3.3 (0.3) | 3.3 (0.1), n.s. |
| **7-Day R** | 3.3 (0.3) | 3.5 (0.1), n.s. |
| **15-Day R** | 3.6 (0.3) | 3.5 (0.1), n.s. |
| **30-Day R** | 3.5 (0.4) | 3.4 (0.2), n.s. |

Data are presented as mean (standard deviation). Food intake in mice of the I or R cohorts were compared with their age-matched non-immobilized control mice, as described in methods.

*Definition of abbreviations*: g, grams; h, hours; I, immobilization; R, recovery.

*Statistical significance*: n.s., non-significant differences between either I or R cohorts of mice and their respective non-immobilized age-matched controls.


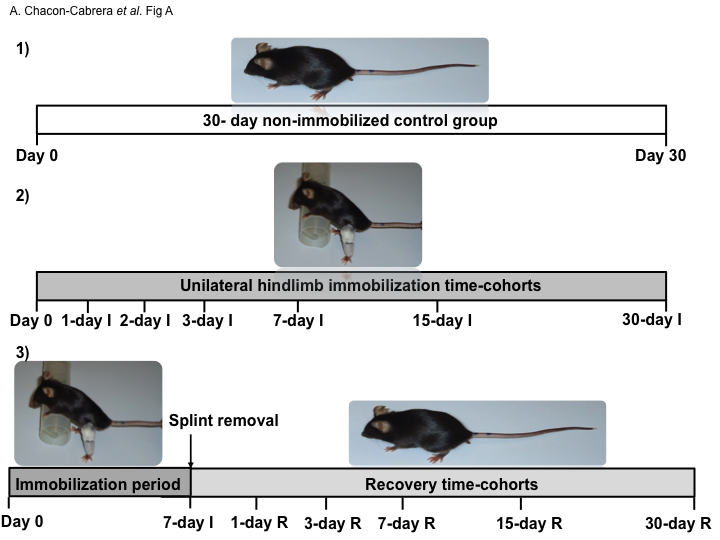


**Fig A.** **1)** 30-day non-immobilized control group, **2)** unilateral hindlimb immobilization time cohorts, and **3)** recovery time cohorts. Definition of abbreviations: I, immobilization; R, recovery.


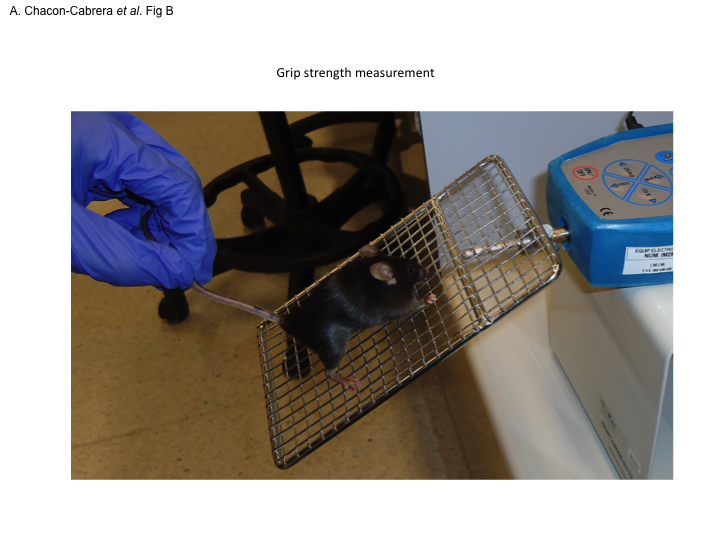


**Fig B.** Grip strength was assessed in the four limbs at the same time in all mice.


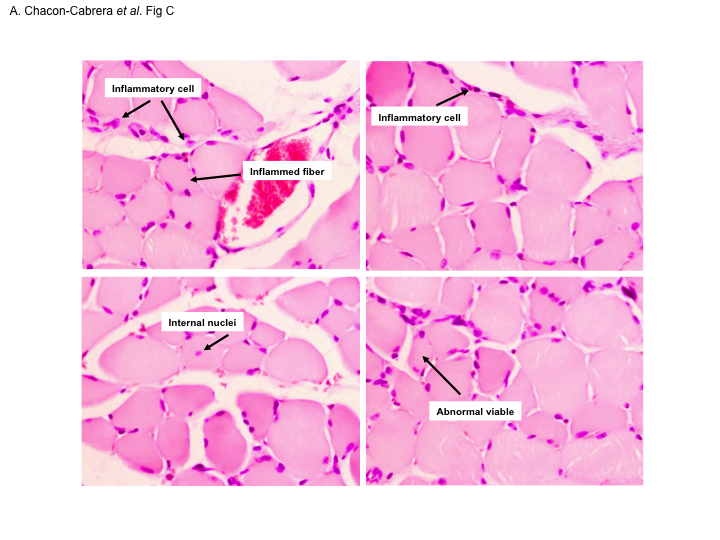


**Fig C.** Representative examples of 4 grids of 63 point-intercepts extracted from a gastrocnemius of immobilized mice (x 400), in which all different categories for point counting were identified. Those categories were defined using a specific number as described in the Methods section above.


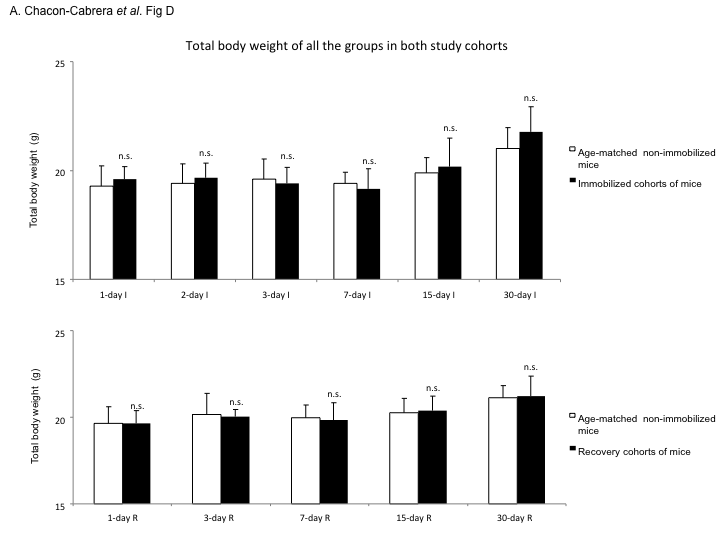


**Fig D.** Mean values and standard deviations of total body weights in mice from the immobilization (top panel) and recovery (bottom panel) groups compared with their age-matched non-immobilized control animals. Definition of abbreviations: g, grams; I, immobilization; R, recovery. Statistical significance is represented as follows: n.s., non-significant differences between either immobilized or recovery groups of mice and the non-immobilized controls.


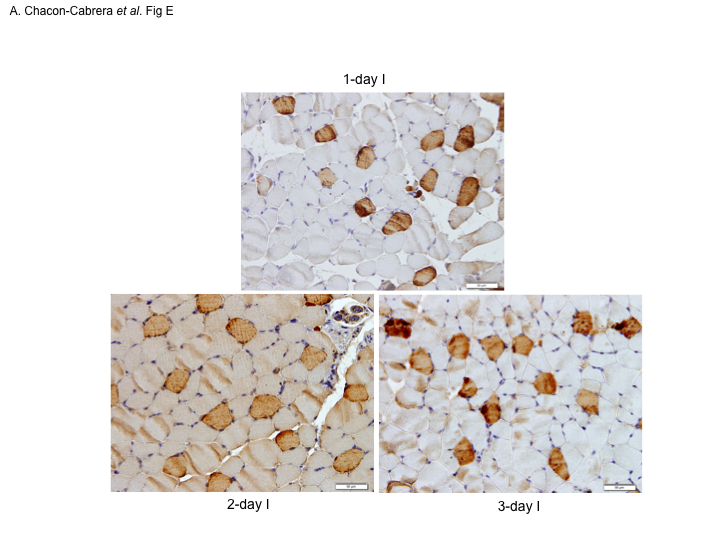


**Fig E.** Representative examples of the gastrocnemius muscle in animals of the immobilized cohorts, and non-immobilized control group. Myofibers positively stained with the anti-MyHC type I antibody are stained in brown color (x 400). Type II fibers were not stained (white color).


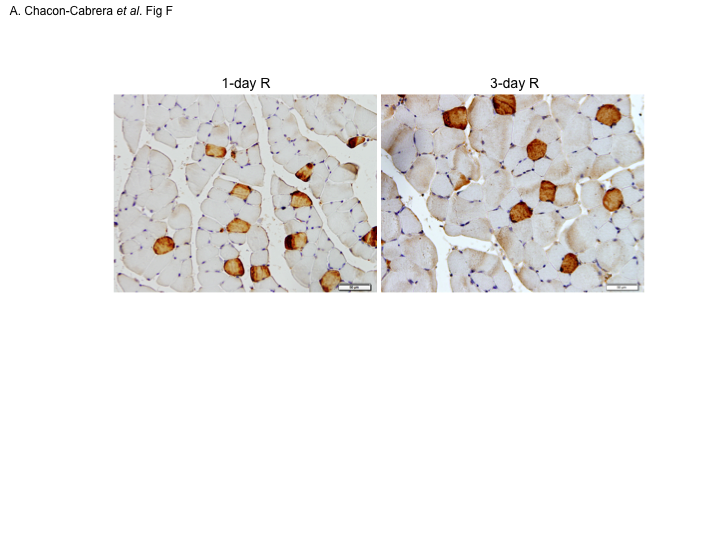


**Fig F.** Representative examples of the gastrocnemius muscle in animals of the recovery cohorts. Myofibers positively stained with the anti-MyHC type I antibody are stained in brown color (x 400). Type II fibers were not stained (white color).


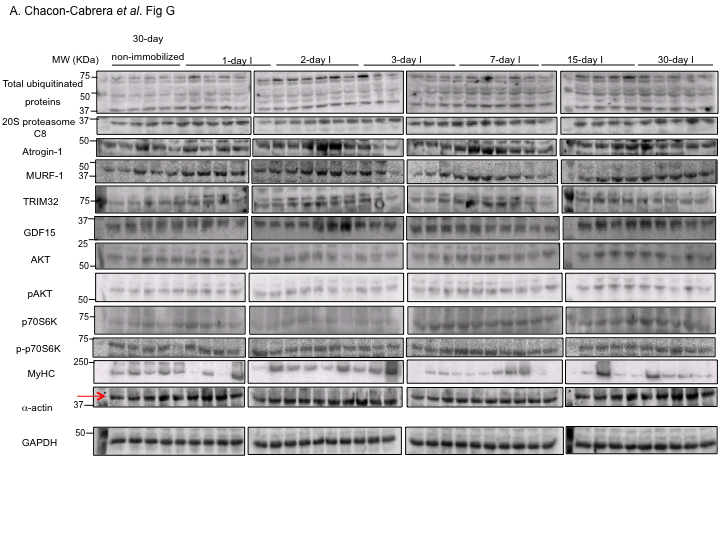


**Fig G.** Immunoblots of markers of proteolysis, muscle anabolism, and muscle structural proteins in the gastrocnemius of the following experimental groups: non-immobilized (N=5), 1-day I (N=5), 2-day I (N=6), 3-day I (N=6), 7-day I (N=6), 15-day I (N=5), and 30-day I (N=5). Representative GAPDH is shown as the loading control. The corresponding molecular weights are indicated for each marker. Definition of abbreviations: I, immobilization; MURF-1, muscle ring finger protein 1; TRIM32, tripartite motif containing 32; GDF15, growth differentiation factor 15; Akt, RAC-alpha serine/threonine-protein kinase; p-Akt, phosphorylated-Akt; p70S6K, p70 S6 kinase; p-70S6K, phosphorylated p70S6K; MyHC, Myosin Heavy Chain; GAPDH, glyceraldehyde-3-phospate dehydrogenase; MW, molecular weight; KDa, Kilodaltons.


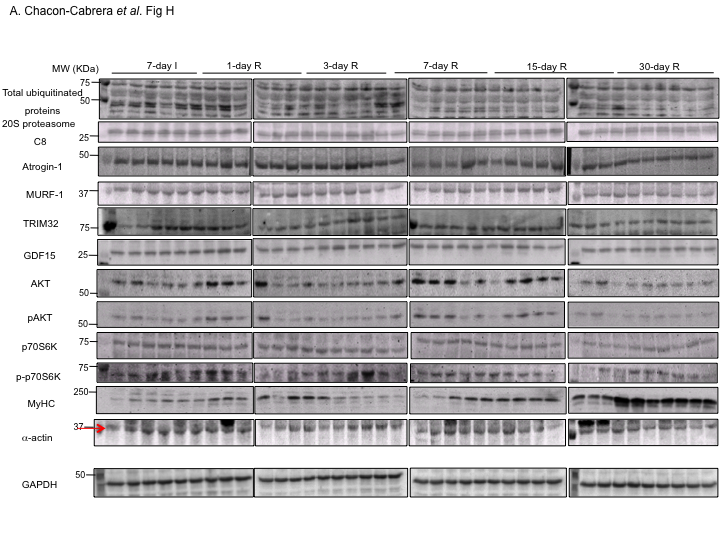


**Fig H.** Immunoblots of markers of proteolysis, muscle anabolism, and muscle structural proteins in the gastrocnemius of the following experimental groups: 7-day I (N=6), 1-day R (N=6), 3-day R (N=6), 7-day R (N=6), 15-day R (N=7), and 30-day R (N=7). Representative GAPDH is shown as the loading control. The corresponding molecular weights are indicated for each marker. Definition of abbreviations: R, recovery; MURF-1, muscle ring finger protein 1; TRIM32, tripartite motif containing 32; GDF15, growth differentiation factor 15; Akt, RAC-alpha serine/threonine-protein kinase; p-Akt, phosphorylated-Akt; p70S6K, p70 S6 kinase; p-70S6K, phosphorylated p70S6K; MyHC, Myosin Heavy Chain; GAPDH, glyceraldehyde-3-phospate dehydrogenase; MW, molecular weight; KDa, Kilodaltons.
